# Scoping Review on Risk Factors and Methods for the Prevention of Bovine Respiratory Disease Applicable to Cow–Calf Operations

**DOI:** 10.3390/ani12030334

**Published:** 2022-01-29

**Authors:** Shih-Yu Chen, Pedro Negri Bernardino, Erik Fausak, Megan Van Noord, Gabriele Maier

**Affiliations:** 1 Department of Population Health and Reproduction, School of Veterinary Medicine, University of California Davis, Davis, CA 95616, USA; msychen@ucdavis.edu; 2 Center for Immunology and Infectious Diseases, University of California Davis, Davis, CA 95616, USA; pnberna@ucdavis.edu; 3 University Library, University of California Davis, Davis, CA 95616, USA; edfausak@ucdavis.edu (E.F.); mgvannoord@ucdavis.edu (M.V.N.)

**Keywords:** bovine respiratory disease, prevention, risk factors, antimicrobial stewardship, shipping fever

## Abstract

**Simple Summary:**

Bovine respiratory disease (BRD) is common in cattle populations and has been named as one of three diseases where antibiotics are most frequently used in cow–calf operations in California. Antibiotics are typically used to treat or prevent the disease, but recent concerns about antibiotic resistance of pathogens in animals or spreading from animals to people have led to the call for more judicious use of these drugs. The present review summarizes the English scientific literature on articles about risk factors for the disease as well as ways to prevent BRD that are applicable to cow–calf operations. Numerous management and animal factors have been identified as increasing the risk for BRD. Vaccinations, metaphylactic use of antibiotics, and feed supplements are areas of research into the prevention of BRD. Genetics have also been explored to determine the heritability of BRD resistance. While vaccinations and metaphylactic use of antibiotics have been evaluated in multiple systematic reviews and meta-analyses, these types of summaries are missing for commonly studied feed supplements, such as yeast and trace minerals, and the use of nitric oxide releasing substance to prevent BRD. Further, it would be beneficial to summarize the knowledge on management related risk factors in literature reviews.

**Abstract:**

The presented scoping review summarizes the available research evidence and identifies gaps in knowledge for bovine respiratory disease (BRD) prevention. Published literature on BRD from 1990 to April 2021 was searched in online databases, including Medline, CAB Abstracts, Scopus, Biosis, and Searchable Proceedings of Animal Conferences. Citations were systematically evaluated in a three-stage approach using commercial software and summarized in a scoping review format. A total of 265 publications were included in this review with herd/farm management (27.9%) as the most prevalent factors studied, followed by metaphylaxis (24.5%), vaccinations (24.1%), diet formulations, and nutritional supplementations (17.7%), animal characteristics (10.2%), and less common interventions and risk factors (6.4%). A high proportion of studies under herd/farm management (73%), metaphylaxis (86%), vaccinations (70%), animal characteristics (78%), and less common interventions and risk factors (59%) showed either significant effects on reducing BRD morbidity or significant differences of BRD between treatments. However, diet and nutritional supplementations reduced BRD only in 30% of research publications. Most studies on BRD were performed in feedlot populations, and more studies in cow–calf populations are needed. We further suggest meta-analyses on the use of yeast and trace mineral supplementation, and nitric oxide-releasing solution for BRD prevention.

## 1. Introduction

Bovine respiratory disease (BRD), also known as shipping fever, is one of the leading causes of illness and death in cattle, with an estimated economic cost of over USD 3 billion annually in the US [1]. Decreases in average daily gain (ADG) ranged between 24–900 g in cattle with BRD compared to healthy cattle in various studies [2,3,4,5].

BRD is a multi-factorial disease complex involving the interaction between environmental factors, host factors, and pathogens. Environmental factors include ambient temperature, humidity, ventilation, dust, ammonia, and overcrowding, while age, sex, weight, nutrition, breed, genetics, immune status, and concurrent disease are host factors related to BRD [6,7]. Viruses, including Bovine Herpesvirus-1 (BHV-1), Bovine Respiratory Syncytial Virus (BRSV), Parainfluenza Virus 3 (PI-3), Bovine Viral Diarrhea Virus (BVDV), and bacteria, such as *Pasteurella multocida*, *Mannheimia haemolytica*, *Histophilus somni*, and *Mycoplasma bovis*, are the main pathogens of BRD [8,9]. In addition to the risk factors above, weaning, commingling, processing, and transportation (shipping) are stressors that are also commonly related to BRD [10].

According to the 2011 National Animal Health Monitoring System (NAHMS) feedlot report, 96.9% and 16.2% of feedlots and cattle were affected by shipping fever, respectively [11]. As for cow–calf operations, approximately 20% of herds reported BRD cases in pre-weaning or nursing calves, and the within-herd BRD prevalence for this population was estimated to be between 5–15% [12,13,14,15]. A USDA report from 2017 found that BRD accounts for 23.9% and 26.9% of nonpredator death loss in cattle and calves, respectively, representing the most common known cause of nonpredator death for cattle [16]. A variety of antimicrobials are licensed for control and treatment of BRD in the US, such as tilmicosin, florfenicol, tulathromycin, danofloxacin, enrofloxacin, and ceftiofur [17]. Metaphylactic, prophylactic, and therapeutic antimicrobials are widely used for prevention and treatment of BRD. In 2016, 41.8% of feedlots used antimicrobials in feed to prevent, control, or treat respiratory disease in the US [18]. However, there is concern that the overuse of antimicrobials in animals may contribute to the development of antibiotic-resistant pathogens that may be transferred to humans. An antimicrobial resistance (AMR) study on *Escherichia coli* O157 from human and animals between 1985 and 2000 indicated that 23.3% of *E. coli* from cattle were resistant, and 71.0% of them were resistant to two or more antimicrobials [19]. Due to the growing concern about AMR, using antibiotics in food-producing animals more judiciously has been legislated by the US Food and Drug Administration (FDA) and other regulatory governmental bodies.

Despite the advantages of antimicrobials, finding alternative ways of preventing and controlling BRD, including identifying the risk factors for BRD, effective vaccination of animals, changing management strategies, and having cattle that are more resistant to BRD, is urgently needed. A large body of research is available in the literature on risk factors and prevention of BRD. A majority of studies were performed in feedlots focusing on transportation as one of the major causes of BRD. As an example, the timing of vaccination with a multivalent modified live virus BRD vaccine of cattle arriving at a feeding facility after commingling and transportation was evaluated in a study, which is typical for this body of literature [20]. 

The most common reasons for the use of antimicrobials in cow–calf operations in California are for the treatment of BRD, infectious bovine keratoconjunctivitis, and digestive diseases according to a survey conducted by the Antimicrobial Use and Stewardship group within the California Department of Food and Agriculture [21]. Transporting cattle from place to place is a very common practice in the cattle industry, either for taking advantage of available feed during different seasons or for specific parts of the production cycle, such as weaning, growing, fattening, or processing [22]. While not all transportation of cattle results in BRD, length of transport, commingling of cattle from the same or other herds, handling, and environmental conditions are risk factors that may enhance the risk of disease. In order to evaluate the available literature on ways to reduce the use of antimicrobials or to use them more judiciously, a scoping review on the prevention of BRD with a focus on transportation was conducted. The objective of the scoping review is to map and summarize the available research evidence and to identify gaps in knowledge or areas with an abundance of studies that would benefit from meta-analyses or systematic reviews.

## 2. Materials and Methods

### 2.1. Protocol and Registration

The preferred reporting items for systematic review and meta-analysis protocols (PRISMA-P) [23] as well as the PRISMA Extension for Scoping Review guidelines [24] were followed for this review. The study protocol has been deposited with the University of California open access publications platform eScholarship on 16 October 2020 [25].

### 2.2. Focus Group and Eligibility Criteria

Prior to the start of the study, a group of bovine practitioners in private practice, academia, and from the California animal health body, together with a veterinary laboratory diagnostician, and an animal science faculty, met to discuss the goals and scope of this review. Outcomes, interventions, and pathogens to be considered for the literature search were discussed. One of the management practices associated with BRD in cow–calf operations identified by the focus group was transportation of cattle throughout the production cycle to take advantage of pasture or rangeland at different times of the year in different geographic locations. Based on the meeting discussions, the following inclusion criteria for literature in this review were established: no geographical limitations; inclusion of relevant studies in feedlots and with dairy breeds; available in English; one or more variables being compared among groups to affect BRD outcomes (case series and reviews were not included), either as interventions (e.g., vaccination), or observed aspects of the animals (e.g., breed), or management (time of transportation); and published in 1990 or later. Due to the large body of work expected to exist on the topic of BRD and to limit the review to the most recent advances in the field, it was deemed appropriate to restrict searches to the last 30 years, although the protocol states a start date of 1950 for publication dates.

### 2.3. Information Source

The literature search was performed by librarians at the Carlson Health Sciences Library at the University of California, Davis. Published literature on BRD was searched in online databases, including Medline (Pubmed interface), CAB Abstracts (CAB Direct interface), and Biosis (Web of Science interface) in July 2020. Common Medical Subject Headings (MeSH terms) were compared across key articles using Yale MeSH analyzer. A follow-up search was performed using Scopus (www.scopus.com (accessed on 23 April 2021)) for the references of all literature included in this review as well as articles citing the literature included (File S1). Gray literature was searched through the Searchable Proceedings of Animal Conferences (S-PAC) database. The full search strategy can be found in the protocol on eScholarship [15].

### 2.4. Selection of Sources of Evidence

With the initial dataset of published scientific articles acquired, two veterinary medical professionals with epidemiological training screened articles in two stages using systematic review software (DistillerSR, Evidence Partners, Ottawa, ON, Canada). Questions for both stages were tested on a random sample of articles (20 for stage 1 and 10 for stage 2). Adjustments to the questions were made, when necessary, to eliminate any ambiguities. Stage 1 questions were: ‘Is the article in English?’ and ‘Does the study compare an intervention for the prevention of BRD in cattle to either placebo or another intervention or does it evaluate risk factors for BRD between exposed and non-exposed cattle?’ The article passed to the next stage if the answers to the two questions were ‘Yes’ or ‘Not clear’. Stage 2 included eight questions where the answers told us if (1) the full text of the study was available; (2) it was published in 1990 or later; (3) it was a case report/series; (4) it focused on one or more variables related to the prevention or risk of developing BRD; (5) the BRD diagnosis was based on clinical diagnosis or pathogen detection; (6) the variable studied could be generalized to cow–calf operations in California; (7) there was a quantifiable outcome included in the study; and (8) it was a publication in a peer-reviewed journal or conference proceeding with more than 500 words. Articles passed stage 2 if the answers to question 3 was ‘No’ and to all other questions was ‘Yes’. Conflicting answers were discussed with the principal investigator before including or excluding that article.

### 2.5. Data Acquisition and Charting

From articles that passed review stage 2, data were extracted by the two reviewers where each reviewer collected data from half of the articles. Data extraction aimed to acquire information related to study characteristics, intervention characteristics, observations performed, and study outcome. Description of each of these aspects extracted from the articles is shown in Table 1.

## 3. Results

### 3.1. Literature Selection and Overall Trends

**Figure 1:**
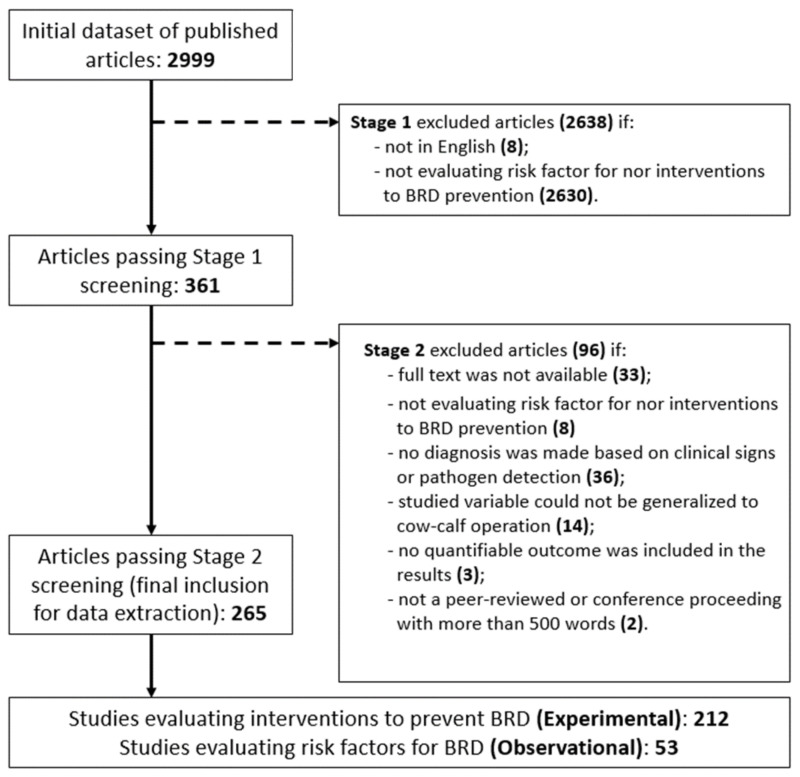
Flowchart depicting the stages for inclusion of published articles related to BRD prevention into a scoping review, showing the reasons for exclusion in each stage.

The search for published literature using the aforementioned tools and search strategies initially resulted in 2999 articles. After stage 1 and 2 evaluations, a total of 265 articles were included in the final review for data extraction of methods to prevent BRD in cow–calf operations File S1. Figure 1 provides the flowchart describing the number of articles acquired initially, together with the reasons for exclusion during stages 1 and 2.

General characteristics of the articles, such as year and location of the study, characteristics of animals used, and overall management features, are described in Table 2 and Table 3. There were more studies published after 2005 (189/265; 71.3%) than from 1990–2005, and in the US or Canada (216/265; 81.5%) than other continents. A minority of articles (less than 50%) did not provide the general characteristics we collected from the studies for every feature evaluated. Beef herds (182/265; 68.7%) and feedlot housing (148/265; 55.8%) were the most prevalent within the selected literature. Categories of animals used in studies with the largest proportions were weaned (139/265; 52.5%), male (104/265; 39.2%), and crossbred (110/265; 41.5%) animals, although when classifying the cattle into crossbred, the specific breeds crossed were not always stated. Among the possible diagnostic methods, the great majority (252/265; 95.1%) diagnosed BRD based on clinical signs, and even when necropsy (32/265; 12.1%) or pathogen detection (28/265; 10.6%) were employed, they were hardly ever made without observation of clinical signs as well. The outcome unit of analysis most frequently used was individual animal count (213/265; 80.4%), and the duration of the studies varied greatly, with a median duration period of 49.5 days, ranging from 6 to 1825 in the day count for follow-up periods.

### 3.2. Herd/Farm Management

**Figure 2:**
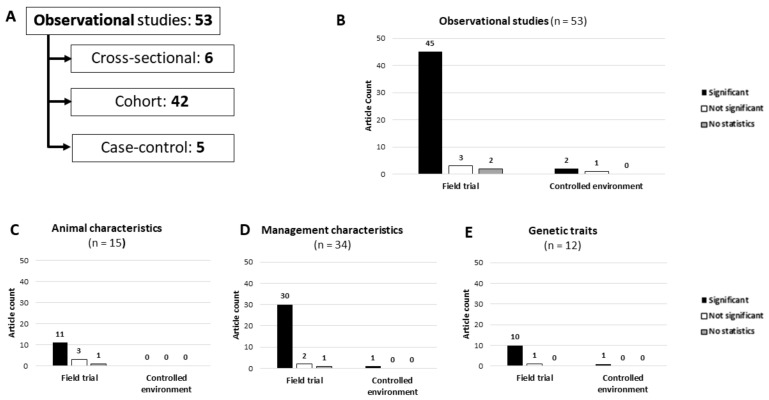
Summary of observational studies included in a scoping review on BRD prevention measures applicable for cow–calf operations. (**A**) Counts of the three main observational study types. (**B**) Number of studies with and without statistically significant results, or studies not reporting statistics. (**C**–**E**) Breakdown of articles based on the main variable(s) under study.

**Figure 3:**
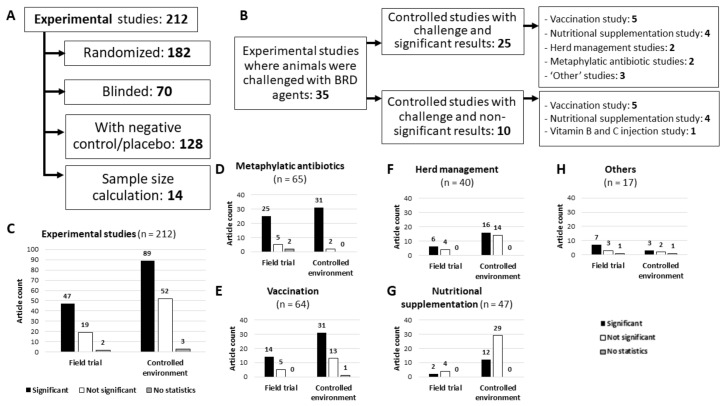
Summary of experimental studies included in a scoping review on BRD prevention measures applicable for cow–calf operations. (**A**) Number of studies that reported features to enhance study quality. (**B**) Number of articles employing challenges with respiratory pathogens. (**C**) Number of studies presenting statistically significant results, non-significant, or studies with no statistics. (**D**–**H**) Breakdown of articles based on the main variable(s) under study.

A large proportion of published literature matching the inclusion criteria of this review studied herd/farm management as variables linked to BRD occurrence (74/265; 27.9%), with 34 observational and 40 experimental studies, as shown in Figure 2 and Figure 3. Among the observational studies, 31 (91.2%) presented statistically significant results, and among the experimental studies, there were 22 (55.0%) with statistically significant results, totaling 53 out of 74 (71.6%) publications indicating that management characteristics play a role in the incidence of BRD. Only 1 (1.3%) publication in this category did not have statistical calculations, and 43 of the 74 (56.6%) were field trials (33 observational and 10 experimental), with 36 of them (83.7%; 30 observational and 6 experimental) presenting outcomes with statistically significant differences among comparisons.

The 34 observational studies looking at herd management analyzed multiple variables that could influence BRD incidence: transportation factors (e.g., rental trucks, time of transportation, animal placement in the truck), environmental factors (e.g., temperature, aerosolized particulate matter), health factors (e.g., weight, lethargic/depressed animals), and other management practices (e.g., herd density, vaccine type and timing, mixing of pens or herds, weaning strategy). Only two (5.9%) showed that those factors did not influence the frequency of BRD significantly, and another one (2.9%) had no statistics calculated, whereas the others (31/34; 91.2%) presented at least one of the factors mentioned above as significantly impacting BRD prevalence. The management practices lumped together as “other” made up most studies in this category, with 21/34 studies (61.7%) showing evidence of effects on BRD incidence, especially mixing of animals (contact amongst pens and introduction of new animals) (10/21; 47.6%), herd size (8/21; 38.1%) and source of animals (4/21; 19.0%). Those studies concluded that allowing cattle from different herds to mix, having large or small herd sizes, or purchasing from sales yards is associated with greater BRD incidence. Nine (26.5%) and six (17.6%) articles correlated health and transport factors, respectively, with BRD frequency, and those showed that poor health history of the herd (presence of BVDV persistently infected, depressed/lethargic animals, or previous infections in the herd), lower body weight, longer transportation, and renting transport trucks could enhance the odds of BRD in the herd. Lastly, only four (11.8%) linked environmental factors with BRD, indicating higher temperatures as the main environmental factor contributing to BRD incidence. Five (14.7%) articles in this category reported sample size calculations for their studies.

A total of 40 experimental studies evaluated herd management features, of which 10 (25.0%) were field trials, with 6 of them presenting statistically significant results (60.0%) indicating that managing weaning stress (2), vaccination timing (2), colostrum management (1), and purchase of castrated animals (1) contribute to lower numbers of BRD cases. The other 30 articles (75.0%) described studies in controlled environments, of which 16 (53.3%) had statistically significant results, suggesting that vaccination (5) and metaphylaxis timing (2), avoiding stress (5), and purchasing animals with health certification (3) are linked to better control of BRD in herds. The studies that failed to show statistically significant differences (4 and 14 for field trials and studies in controlled environments, respectively) considered vaccination timing and route (9), stress control (4), and other practices less commonly associated with BRD (5), such as glycerin drench and steroid implantation timing. Only 13 of the experimental studies (32.5%) mentioned blinding of researchers; 4 of the 13 were field trials with statistically significant results, with 2 also using a negative/placebo control and randomization (studying weaning stress management, and methaphylaxis timing), only 1 randomized (studying colostrum management) and only 1 used a negative/placebo control (studying vaccination timing). There were nine non-field trials that mentioned blinding, seven of which also used randomization, three mentioning sample size calculations, and four of which also included a negative/placebo control, where one failed to show statistically significant differences in vaccination timing, and eight showed statistically significant differences for vaccination and metaphylaxis timing, parasite control with injectable medication, lowering stress, and purchasing calves with health certification for prevention of BRD.

### 3.3. Antimicrobial Metaphylaxis

There were 65 (24.5%) articles evaluating the use of metaphylactic antimicrobial therapy to prevent BRD, all of which were experimental (observational studies on timing of metaphylaxis were classified into management studies), with 32 (49.2%) field trials of which 25 (78.1%) presented statistically significant favorable outcomes for animals receiving antimicrobial metaphylaxis, 5 (7.7%) showing no statistically significant differences, and 2 did not report statistical calculations. Of the studies on metaphylactic antimicrobial therapy that were not field trials (33; 50.8%), all presented statistical analyses, with 31 (93.9%) showing statistically significant favorable outcomes for animals receiving metaphylaxis.

In the 32 field trials, multiple studies compared the use of two or more antimicrobials as prophylaxis/metaphylaxis (see Table 4). Of the 25 field trial articles presenting statistically significant advantages of metaphylactic use, all reported randomization and 10 were blinded studies, which supported the use of tulathromycin (TUL) (3), oxytetracycline (OXY) (2), gamithromycin (GAM) (2), tilmicosin (TIL) (1), ceftiofur (CE) (1), and chlortetracycline (CLO) (1) to prevent BRD, and 3 (9.4%) also reported a sample size calculation comparing OXY (2) and TIL (1) to no metaphylaxis.

The 33 articles describing studies in controlled environments (non-field trials) evaluated the effect of 6 different antibiotics to prevent BRD. Of the 13 articles (39.4%) in this category studying TIL, all showed improved BRD outcomes to using this antimicrobial compared to a control, 2 showed favorable BRD outcomes when used after arrival at the farm compared to before arrival, and 1 found a high dose (20 mg/kg) yielding improved BRD outcomes versus a low dose; however, 1 article reported decreased productivity when using TIL for metaphylaxis. TUL use was the focus of 11 articles (33.3%), where all showed improved BRD outcomes with TUL use compared to negative controls, and 7 compared to TIL, 2 compared to CEF, 2 compared to GAM, and 1 compared to FLO. All studies on the sole use of GAM (three), CEF (one), and FLO (one) presented results supporting their metaphylactic use compared to a control. Studies evaluating tetracycline (5/33, 15.1%; OXY and CLO) had disagreements, with 3 indicating it significantly helped in the prevention of BRD and 2 indicating otherwise. Only two studies challenged animals with *Histophilus somni*, and both showed that antimicrobial metaphylaxis (with TIL or GAM) led to better BRD outcomes than no metaphylaxis.

### 3.4. Vaccination

Vaccination was the third most studied category of interventions among the literature included in this review, with 64 studies (24.1%), all of which were experimental studies. Observational studies on vaccination timing or re-vaccination were categorized into ‘herd/farm management’. Only 19 of the studies on vaccination (29.7%) were field trials, with 14 (73.7%) showing statistically significant differences between study groups. The majority were non-field trials controlling for one or more external variables (45/64; 70.3%) where 31 (68.9%) publications showed statistically significant differences in the results, 13 (28.8%) articles reporting no significant differences, and 1 (2.2%) not reporting calculated statistics. Studies on vaccinations was the category that performed the highest number of challenge infections in study animals (26/64; 40.6%).

We divided vaccines into three main groups of target agents: common BRD viral agents, or cVIR (BVDV, BPI-3, BHV-1, and BRSV), with 40 studies (62.5%); common BRD bacterial agents, or cBAC (*H. somni*, *M. bovis*, *M. haemolytica* (including *P. haemolytica*), *P. multocida*), with 27 studies (42.2%); and agents uncommonly associated with the BRD complex, or ucBRD (*E. coli*, *Salmonella typhimurium*, Coronavirus, Rotavirus, and Influenza virus), with 5 studies (7.8%).

In the cVIR group, there were 11 field trials (27.5%)—all testing commercial vaccines and only 2 not using a placebo group—with 8 of them (72.7%) showing significant differences supporting the use of vaccines to control BRD. Two (18.2%) articles in this group mentioned how sample sizes were derived and two studies reported both randomization and blinding, including one that challenged animals with BHV-1 after immunization. The remaining three field trials (27.3%) did not show statistically significant differences between groups. The 29 articles (72.5%) on cVIR vaccines of studies conducted in controlled environments included 19 (65.6%) showing statistically significant differences between treatments (17 commercial and 2 experimental vaccines, 10 challenged with BHV-1, BPI-3, or BRSV), 9 (31.0%) showing no statistically significant differences between treatments (8 commercial and 1 experimental vaccine, 2 challenged with BHV-1 or BPI-3), and 1 (3.4%) not reporting statistics (using a commercial vaccine).

The cBAC studies had nine field trials (33.3%), of which six (66.7%) presented statistically significant differences between study groups (four commercial and two experimental vaccines, one using randomization, blinding, and a placebo group for a commercial vaccine against *P. haemolytica,* and one challenged animals with *M. haemolytica* after using a commercial vaccine). Two (22.2%) articles also reported a sample size calculation. Of the 18 non-field trials using cBAC (66.7%), 13 reported statistically significant differences (72.2%) (8 commercial and 5 experimental vaccines, 5 challenged with one or multiple bacterial agents of which 2 had used commercial vaccines). The five non-field trials (27.8%) that failed to report statistically significant differences between groups included three commercial and one experimental vaccine—this last one used before a challenge with *H. somnus*—as well as one study with no statistics calculated for an experimental vaccine against *M. bovis*.

The last group (ucBRD) included two (40.0%) field trials showing that vaccination lowers the BRD incidence, with both using commercial vaccines, a placebo group, and randomization, but only one reporting blinding of researchers. The other three (60.0%) were studies in controlled environments with two (67.7%) reporting results of significantly decreasing BRD rates in vaccinated animals—including one study challenging animals with the Influenza virus and using an experimental vaccine against the same agent.

### 3.5. Diet Formulation and Nutritional Supplementation

Among the 265 publications included in this review, 47 (17.7%) evaluated the impact of different diets and supplementations on the occurrence of BRD, all of which were experimental. This category showed the least number of articles with statistically significant differences (14/47; 29.8%), and also included the fewest field trials (6/47; 12.8%), where the majority did not show statistically significant differences (4 studies evaluating immune primer formulas, vitamin E supplementation, or different diet formula). Only two articles in this category reported statistically significant results, with both employing randomization and a negative control, but only one blinding the personnel collecting data (studying melengestrol acetate supplementation) and the other not (studying chromium supplementation). Only one (2.1%) study in this category reported a sample size calculation.

The other 41 studies (87.2%) were conducted in controlled environments, of which 12 reported statistically significant results and used negative controls. Four of these articles also reporting blinding and randomization studied yeast supplementation (two), a commercial nutritional mix (one), melengestrol acetate feeding and challenge with *M. haemolytica* (one). Five articles reported only randomization of groups and studied yeast supplementation (two), and different diet formulas/supplementation mixes (three)—one of them challenging with BHV-1. Three articles reported neither blinding nor randomization and studied supplementation with immune primer formulas (one) and yeast supplementation (two), with one of them challenging with LPS. The remaining 29 articles did not show statistically significant differences and studied a variety of different diet formulations, nutritional or immune primer mixes, vitamins and minerals, or yeast supplementation.

### 3.6. Animal Characteristics

This category can be divided into two classes according to the variable being considered in the publication. Out of the 27 studies (10.2%), there were 15 evaluating the animal’s phenotypes (55.5%), and 12 the animal’s genetics (44.5%). All were observational studies, and only one (3.7%) was not a field trial (studying genetic traits related to BRD).

From the 15 articles evaluating the animals’ phenotypes as a variable linked to BRD, 3 (20.0%) did not show statistically significant differences, and only 1 (6.7%) did not calculate statistics, but it showed higher incidence of BRD in younger animals (<105 days old). The other 11 (73.3%) presented statistically significant differences and all evaluated multiple animal characteristics. A sample size calculation was mentioned in one (13.3%) article. Higher BRD occurrence was associated with older animals (3/11; 27.3%; age range was not the same across studies), male sex (4/11; 36.3%), lower body weights (2/11; 18.1%), and Hereford breed (4/11; 36.3%). Other characteristics evaluated were dentition (as age estimation), region of origin, chest circumference (as weight estimation), season of birth, and bloodline.

From the 12 publications about the animals’ susceptibility to BRD due to genetics, 11 (91.7%) were field trials. One (8.3%) did not find any genetic trait linked to BRD, while the other 11 (91.7%) were able to correlate various specific genes with the occurrence of BRD in the animals or calculate the heritability of resistance to BRD. A sample size was calculated in one (8.3%) article.

### 3.7. Miscellaneous Interventions and Risk Factors

Lastly, there were 17 studies (6.4%), all experimental, evaluating if some interventions, not commonly associated with BRD control or not commonly employed in cow–calf operations, could help in the prevention of BRD. There were 10 presenting statistically significant results (58.8%), and of those 3 showed higher BRD prevalence in the groups using the novel intervention studied (aspirin, bovine transfer factor or nitric oxide), while the other 7 presented lower BRD counts in the groups with the novel intervention (serum from vaccinated animals, nitric oxide, natural human interferon-alpha, oxfendazole associated with fenthion, injection of trace minerals, and liposome with toll-like receptors 3 and 9 agonists). Two studies (11.7%) did not calculate statistics but showed a higher incidence of BRD in animals using nitric oxide instead of TIL, and synergy between BRSV and 3-methylindole to cause BRD. The other five (39.4%) showed no statistical difference among groups when comparing a negative/placebo control with the studied interventions (nitric oxide, meloxicam, injection of vitamin B and C or trace minerals, and growth implants).

## 4. Discussion

This scoping review summarized the available research evidence on the prevention of BRD with an emphasis on BRD following transportation, which is a risk factor in some cow–calf operations. There are systematic review or meta-analysis articles describing specific prevention methods for BRD, including antimicrobial use and vaccination [26,27,28]. However, to our knowledge, no meta-analysis, scoping, or systematic review papers have been published on integrative BRD prevention.

The presence of BRD-related pathogens alone does not necessarily mean that the animal is sick because many of these pathogens are opportunistic, and disease occurs only as a multi-factorial disease complex. Therefore, we only included studies with disease (BRD or undifferentiated fever) as an outcome and excluded those that only detected specific antibodies or pathogens. Most studies related to BRD prevention were published after 2005, and most of them were from the US or Canada, which is not unexpected because the US has the largest cattle industry in the world [29]. Studies focused on beef cattle were the most prevalent within the selected literature. Transportation of cattle is a common husbandry activity in the beef industry, and beef cattle are often shipped multiple times during different stages of production, such as from cow–calf to stocker or backgrounding facilities, and more studies were available on shipping fever in beef cattle [10,30,31]. BRD was mainly diagnosed based on clinical signs. Even though necropsy is the most accurate diagnostic, this method is costly and can only be used on a dead animal. Although clinical observation as a diagnostic measure is flawed, it is the most common way to diagnose BRD in the field.

Different study designs are aimed to analyze predictors from different aspects: observational studies generally analyze multiple factors at the same time in a field setting where adjustment of confounders is addressed during data analysis; experimental study designs, on the other hand, usually focus on specific predictors or interventions controlling for other non-target factors in the study design. The definition of field trial in this study was studies that were conducted on commercial farms and did not challenge animals with BRD pathogens, whereas controlled environment studies were those that were conducted in research facilities or that challenged animals with BRD pathogens. Field trials represent the natural exposures and actual circumstances of husbandry. Experimental field trials controlling for possible confounders through randomization and avoiding biases through blinding of researchers result in more accurate estimates for the effect of a factor, and thus this type of study provides the strongest evidence.

Most observational studies included in this review were field trials, while most experimental studies were conducted in a controlled environment. Observational studies in this review included cohort, cross-sectional, and case-control studies. There were two studies we classified as conducted in controlled environments rather than field trials: one study used data from four previously conducted experimental studies, where the exposures under study were not the same as in the original studies and was categorized as a retrospective cohort study [32]. Another study exposed all cattle to IBR and subsequently analyzed differences in genomic DNA makeup, and was also categorized as a retrospective cohort study design. Observational studies generally analyzed many factors, where often one or more showed to be significantly associated with BRD, while in experimental studies that were focused on few specific factors, statistically significant differences were not always obtained.

Quality assessment of articles is not part of a scoping review; however, it should be noted that many experimental studies included in this review lacked mention of randomization, blinding, the use of a placebo, or sample size calculations, which has also been remarked by the authors of several of the systematic reviews and meta-analysis studies referenced.

Although the aim of the scoping review was to summarize evidence for the prevention measures for BRD applicable to cow–calf operations with an emphasis on transportation, we did not preclude articles conducted in weaned or feedlot cattle or dairy cattle, because there would have been very few articles left. Clearly, not all the evidence summarized here may be translatable to cow–calf herds directly; however, trends apparent from this review and others may still provide valuable information on where to focus efforts for BRD reduction. Interventions that required direct handling of animals more than twice were not considered for inclusion because they were not considered applicable to cow–calf operations.

The most common studies were related to herd/farm management, which included transportation factors (rental trucks, time of transportation, and animal placement in the truck), environmental factors (temperature and aerosolized particulate matter), health factors (induction weight and lethargic/depressed animals), and other management practices (herd density, vaccine type and timing, metaphylaxis timing, and mixing of pens or herds). Analysis of management characteristics showed that the risk of BRD was significantly higher for lighter calves after being introduced to a new environment or when a water source was shared between pens. BRD was also significantly associated with commingling cattle from different sources, shipping distance, or season of induction. Herd/farm management had effects on BRD in more than three-quarters of selected studies. There is a systematic review on vaccination timing, which suggested no difference between vaccines used at arrival or after a delay in terms of morbidity, retreatment risk, and mortality of BRD [33]. However, systematic reviews that analyze all management characteristics or even specific factors within this category may not be feasible. Take weaning stress as an example; three studies were related to weaning time, while another three studies analyzed different types of weaning practices, i.e., yard weaning, pasture/truck/drylot weaning, and preconditioned weaning respectively. Comparing these studies in a systematic review is hindered by varying study designs and outcomes assessed. However, as suggested by Wisnieski et al. [34], predictive modeling of factors associated with BRD in these studies may still serve to produce useful tools, such as best management practices, to reduce BRD in various scenarios.

Metaphylaxis is the most studied intervention in our review for experimental studies, and much of this research has focused on arrival at feedlots. In cow–calf operations, metaphylactic antimicrobial treatments are not commonly applied, likely because of costs and the cost-effectiveness of these treatments in cow–calf herds is not well studied. A study published in 1995 found that metaphylactic tilmicosin injection did not significantly reduce the incidence of BRD in nursing beef calves [35]. Although metaphylaxis is not a common practice in cow–calf operations, studies are still needed to evaluate the usefulness of metaphylaxis under certain conditions, e.g., stressful events, transportation of herds, intensively managed herds that experience outbreaks. A higher percentage of studies on metaphylaxis significantly reduced BRD in controlled environments than in field trials in this review, which may be the case because fewer factors are being controlled in field trials and an effect may be masked by confounding factors. Several recent systematic reviews on metaphylaxis for BRD are available. One such study concluded that using oxytetracycline or tilmicosin on arrival at feedlot reduces BRD morbidity rate [26]. A second meta-analysis of metaphylaxis treatments for BRD found decreased odds of BRD morbidity in beef cattle treated with a metaphylactic antimicrobial compared to non-treatment [36]. A further systematic review of injectable antibiotics for the control of BRD in feedlot cattle was published recently in 2019 [28]. The review suggested that macrolides were the most effective antibiotics to reduce the incidence of BRD within 45 days of feedlot arrival, but because injectable oxytetracycline is already commonly used and is also effective, its use might offer advantages in antimicrobial stewardship for BRD control. However, another systematic review and meta-analysis of randomized clinical trials stated that metaphylaxis should be re-evaluated because, although antimicrobial mass medications relatively lowered disease burden, it did not economically reduce the absolute risk for displaying clinical signs of BRD or having further antibiotic treatments [30]. In addition, concern was expressed by the authors about the use of mass medicating with clinically important antimicrobials.

About three-quarters of studies in the vaccination category were performed in controlled environments because many included a pathogen challenge, which may be followed by field trials if vaccines seem promising. Over two-thirds of selected studies supported the use of vaccines to control BRD in this review. However, vaccines may not always have the desired efficacy. A systematic review and meta-analysis study of commercially available vaccines found that the use of vaccines at or near arrival at feedlot did not reduce the incidence of BRD [27]. There were another two systematic review and meta-analysis studies on viral and bacterial vaccines for BRD, respectively. The viral vaccine review evaluated the effectiveness of commercially available vaccines, showing a significantly decreased BRD morbidity in beef calves vaccinated with various antigen combinations compared to non-vaccinated calves [37]. This study also suggested that BHV-1 and modified live virus (MLV) BVDV vaccines have an effect on reducing BRD morbidity, while cattle vaccinated with MLV BRSV and PI3 vaccines did not have significantly lower BRD morbidity or mortality compared to non-vaccinated calves. A systematic review on bacterial BRD vaccines indicated that *M. haemolytica* and *P. multocida* vaccines are beneficial for decreasing BRD in feedlot cattle, but no evidence was found that *H. somni* vaccines could reduce the incidence of BRD [38]. Overall, there is overwhelming support for vaccination for respiratory disease in calves, e.g., from the American Association of Bovine Practitioners’ vaccination guidelines [39]. However, under certain circumstances, the effects of vaccination on BRD may be reduced, such as immunosuppression or poor body condition. Shipping is a major stressor that could lower the immunity, and therefore, at arrival, vaccination, which is also a stressful practice for cattle, may not have protective effects or may even increase the risk of BRD. The increased risk of BRD in cattle with on-arrival vaccination compared to non-vaccination was observed in a randomized controlled trial [40]. The effects of vaccination may be different in cow–calf herds, but there were limited related studies in this population. Systematic reviews on vaccination for BRD may not be needed at present as several such studies are available, but we suggest more studies on vaccination for BRD prevention in cow–calf operation cattle.

About 69% of studies under the diet and supplementation category did not have significant results to support that diet formulation and nutritional supplementation had an effect on reduction of BRD, which was supported by a review article that the effects of diet and nutritional supplementation on decreasing BRD were not always promising [41]. Nutritional supplementation may not have a direct effect on BRD, but it may improve the performance of other interventions, such as antimicrobials on BRD. For example, a study found that a yeast product did not decrease BRD morbidity by itself, but significantly reduced BRD in heifers that received florfenicol on arrival compared to those receiving only florfenicol [42]. To our knowledge, there were no systematic reviews or meta-analysis studies on diet and nutritional supplementation for BRD prevention. Based on the selected literature in this review, yeast and trace mineral supplementation seemed to have potential benefits of reducing BRD. All of the studies for yeast (10 articles) and trace minerals (8 articles) were randomized clinical trials with morbidity as an outcome. Therefore, we suggest systematic reviews and meta-analyses on these two nutritional supplementations for BRD prevention to further evaluate their efficacy.

Animal characteristics contain many factors, such as age, sex, breed, body weight, and region of origin, and these factors may be most useful in characterizing risk factors for BRD since they do not offer any possibility for intervention. Genetic selection is another solution that could be exploited to reduce BRD morbidity. Heritability estimates for resistance to BRD were reported as low to moderate within the selected studies, which indicated that genetic selection may have potential to select animals that are more resistant to BRD. A genetics review article early in 2009 concluded that the heritability estimates for resistance to BRD were small, but two reviews recently published in 2020 revealed low to moderate heritability estimates [43,44,45]. These two studies also suggested that genetic selection could be utilized to enhance resistance to BRD.

Systematic reviews for less common interventions and risk factors may not be feasible because of the variety of factors studied, except for nitric oxide-releasing solution (NORS). There were four studies on preventing BRD by nasal instillation of nitric oxide-releasing solution with the same study design and outcomes of interests; therefore, we suggest a systematic review and meta-analysis on the use of NORS for BRD prevention.

## 5. Conclusions

In this systematic review on prevention measures for BRD with an emphasis on transportation and applicable to cow–calf operations, the most prevalent studies were related to herd/farm management, followed by metaphylaxis, vaccinations, diet formulations and nutritional supplementations, animal characteristics, and less common interventions and risk factors. Based on the selected studies, the advantages of metaphylaxis have been well documented, but antimicrobial mass medication is also debated due to cost-effectiveness in low-risk herds, and the use of clinically important antimicrobials for this purpose. Vaccinations have shown benefits under the right circumstances. Diet and nutritional supplements seem to have limited efficacy but may be able to enhance immunity. Overall, most studies on this topic were performed in feedlot populations as the cattle production type where BRD is most commonly diagnosed. Although some of the evidence may translate to cow–calf populations, we suggest more studies in this production class because conditions are different and there is enough morbidity due to BRD that merits further research. We further suggest meta-analyses in the areas of the use of yeast and trace mineral supplements, as well as NORS administration for BRD prevention.

## Figures and Tables

**Table 1:** Description of items extracted in the data acquisition step of 265 relevant journal articles or conference proceedings for a scoping review on risk factors and methods to prevent bovine respiratory disease (BRD) in cow–calf operations.

Variable	Description of Items
Study Characteristics	Year of publication; publication type (peer-reviewed journal or conference abstract); region of the study (USA or Canada, Latin America, Europe, etc.); herd type (beef or dairy); housing type (feedlot, pasture, etc.); age of animals (weaned or pre-weaned); sex of animals (male or female); breed of animals (Angus, Holstein, Charolais, etc.); BRD diagnosis (clinical signs, necropsy, and pathogen detection); unit of analysis (individual or herd); number of animals/herds; follow-up period in days; field trial study (yes or no); study type (experimental or observational)
Intervention Characteristics	Overall characteristics (randomized, blinded, had control/placebo, or reported sample size calculation); intervention type (vaccination, management, metaphylaxis, etc.); type of vaccine (attenuated, inactivated, etc.); pathogen in vaccine (BVDV, PI-3, etc.); antibiotic used (tulathromycin, tilmicosin, etc.); supplementation used (yeast, immune formulas, etc.); material type (commercial or experimental); management (castration, stress, etc.); infection challenge; challenge pathogen (BVDV, Influenza, etc.)
Observations Performed	Type of observation (cross-sectional, case-control, or cohort); risk factors studied (animal characteristics, management characteristics, and genetic traits)
Study Outcome	Final result (no statistically significant difference, statistically significant difference, etc.); aspect evaluated for final result (morbidity, mortality, risk factors for BRD)

**Table 2:** Publication characteristics of 265 articles studying BRD prevention, included in a scoping review on the prevention for BRD applicable to cow–calf operations.

Characteristic	Count of Articles	Frequency (%)
Publication year		
1990–1995	29	10.9
1996–2000	19	7.2
2001–2005	28	10.6
2006–2010	64	24.1
2011–2015	52	19.6
2016–2021	73	27.6
Publication type		
Peer-reviewed journal	262	98.9
Conference abstract	3	1.1
Region study was performed		
USA or Canada	216	81.5
Europe	26	9.8
Latin America	3	1.1
Asia	7	2.6
Oceania	12	4.5
Africa	1	0.4

**Table 3:** Population and study characteristics of 265 articles studying BRD prevention, included in a scoping review on the prevention for BRD applicable to cow–calf operations.

Characteristics	Count of Articles	Frequency (%)
Herd type		
Beef	182	68.7
Dairy	26	9.8
Beef and Dairy	8	3.0
Not stated	49	18.5
Housing type ^1^		
Feedlot	148	55.8
Pasture	7	2.6
Pens (not within feedlot)	56	21.1
Individual housing	11	4.1
Not stated	51	19.2
Animals’ age		
Pre-weaned	23	8.7
Weaned	139	52.5
Pre-weaned and Weaned	16	6.0
Not stated	87	32.8
Animals’ sex		
Male	104	39.2
Female	38	14.2
Male and Female	64	24.1
Not stated	59	22.2
Animals’ breed ^1^		
Angus	4	1.5
Holstein	16	6.0
Charolais	2	0.8
Crossbreed	110	41.5
Mixed breeds	34	12.8
Others	5	1.9
Not stated	94	35.5
Diagnostic method ^1^		
Clinical signs	252	95.1
Necropsy findings	32	12.1
Pathogen detection	21	7.9
Unit of analysis		
Individual	213	80.4
Herd	47	17.7
Individual and Herd	5	1.9
Follow-up period (in days)		
Median	49.5	
Range	6–1825	

^1^ Articles may have been counted in more than one category.

**Table 4:** Field trial studies on metaphylactic use of antimicrobials included in a scoping review on the prevention of BRD applicable to cow–calf operations.

Antimicrobial Used for Metaphylaxis	Comparison Group Received	Number of Studies ^1^	Outcome
Tilmicosin	No metaphylaxis	8	Statistically significantly fewer BRD cases than comparison group
	Oxytetracycline	2	
Tulathromycin	No metaphylaxis	1	
	Tilmicosin	2	
	Oxytetracycline	2	
	Gamithromycin	1	
Gamithromycin	Tilmicosin	1	
	No metaphylaxis	3	
Ceftiofur	Tilmicosin	1	
Oxytetracycline	No metaphylaxis	4	
Florfenicol	No metaphylaxis	1	
Chloretracycline	No metaphylaxis	1	
Tilmicosin	No metaphylaxis	2	No statistics provided
Oxytetracycline	No metaphylaxis	1	No statistically significant differences between groups
	Tilmicosin	1	
Tulathromycin	Forfenicol	1	
Gamithromycin	Tilmicosin	1	
Florfenicol	No metaphylaxis	1	

^1^ Articles may have been counted in more than one category.

## Data Availability

The study protocol is available at https://escholarship.org/uc/item/2t11z0tg (accessed on 29 December 2021). Data included can be made available by contacting the corresponding author at gumaier@ucdavis.edu.

## Supplementary Materials

The following are available online at https://www.mdpi.com/article/10.3390/ani12030334/s1, File S1: References Included in Scoping Review.
