# Supplementary material for: Scoping Review on Risk Factors and Methods for the Prevention of Bovine Respiratory Disease Applicable to Cow–Calf Operations
Source: Animals (Basel). 2022 Jan 29;12(3):334. doi: 10.3390/ani12030334 (PMC8833575; doi:10.3390/ani12030334)
Supplement: Supplementary file 1 [file animals-12-00334-s001.zip › animals-1561592-supplementary.pdf]

Supplement S1 to article in Animals "Scoping Review on Risk Factors and Methods for the Prevention of Bovine Respiratory Disease Applicable to Cow-Calf Operations"

Shih-Yu Chen, Pedro Negri Bernardino, Erik Fausak, Megan Van Noord, and Gabriele Maier

References included in scoping review. Categories are not mutually exclusive such that a reference may appear more than once.

Herd/farm management:

1. Ribble, C.S.; Meek, A.H.; Janzen, E.D.; Guichon, P.T.; Jim, G.K. Effect of time of year, weather, and the pattern of auction market sales on fatal fibrinous pneumonia (shipping fever) in calves in a large feedlot in Alberta (1985-1988). *Can J Vet Res*, **1995**; 59(3), pp. 167-172.
2. Ribble, C.S.; Meek, A.H.; Shewen, P.E.; Jim, G.K.; Guichon, P.T. Effect of transportation on fatal fibrinous pneumonia and shrinkage in calves arriving at a large feedlot. *J Am Vet Med Assoc*, **1995**; 207(5), pp. 612-615.
3. Ribble, C.S.; Meek, A.H.; Shewen, P.E.; Guichon, P.T.; Jim, G.K. Effect of pretransit mixing on fatal fibrinous pneumonia in calves. *J Am Vet Med Assoc*, **1995**; 207(5), pp. 616-619.
4. Van Donkersgoed, J.; Klassen, P. Serological study of a modified-live virus IBR vaccine given to feedlot calves after arrival. *Can Vet J*, **1995**; 36(6), pp. 394.
5. Duff, G.C.; Malcolm-Callis, K.J.; Walker, D.A.; Wiseman, M.W.; Galyean, M.L.; Perino, L.J. Effects of intranasal versus intramuscular modified live vaccines and vaccine timing on health and performance by newly received beef cattle. *Bov. Pract.*, **2000**; 34(1), pp. 66-71.
6. Duff, G.C.; Walker, D.A.; Malcolm-Callis, K.J.; Wiseman, M.W.; Hallford, D.M. Effects of preshipping vs. arrival medication with tilmicosin phosphate and feeding chlortetracycline on health and performance of newly received beef cattle. *J Anim Sci*, **2000**; 78(2), pp. 267-274.
7. Norström, M.; Skjerve, E.; Jarp, J. Risk factors for epidemic respiratory disease in Norwegian cattle herds. *Prev. Vet. Med.*, **2000**; 44(1-2), pp. 87-96.
8. Frank, G.H.; Duff, G.C. Effects of tilmicosin phosphate, administered prior to transport or at time of arrival, and feeding of chlortetracycline, after arrival in a feedlot, on Mannheimia haemolytica in nasal secretions of transported steers. *Am J Vet Res*, **2000**; 61(12), pp. 1479-1483.
9. Kerkhofs, P.; Renjifo, X.; Toussaint, J.F.; Letellier, C.; Vanopdenbosch, E.; Wellemans, G. Enhancement of the immune response and virological protection of calves against bovine herpesvirus type 1 with an inactivated gE-deleted vaccine. *Vet. Rec.*, **2003**; 152(22), pp. 681-686.
10. Assié, S.; Seegers, H.; Beaudeau, F. Incidence of respiratory disorders during housing in non-weaned Charolais calves in cow-calf farms of Pays de la Loire (western France). *Prev. Vet. Med.*, **2004**; 63(3-4), pp. 271-282.
11. Ellis, J.; Waldner, C.; Rhodes, C.; Van, R. Longevity of protective immunity to experimental bovine herpesvirus-1 infection following inoculation with a combination modified-live virus vaccine in beef calves. *Journal of the American Veterinary Medical Association*, **2005**; 227(1), pp. 123-128.

12. O'Connor, A.M.; Sorden, S.D.; Apley, M.D. Association between the existence of calves persistently infected with bovine viral diarrhea virus and commingling on pen morbidity in feedlot cattle. *Am J Vet Res*, **2005**; 66(12), pp. 2130-2134.
13. Burciaga-Robles, L.O.; Step, D.L.; Holland, B.P.; McCurdy, M.P.; Krehbiel, C.R. Effect of Castration Upon Arrival on Health and Performance of High Risk Calves During a 44 day Receiving Period. *Proceedings of the 2006 Annual Convention of the American Association of Bovine Practitioners*, **2006**; 39, pp. 234-235.
14. Lago, A.; McGuirk, S.M.; Bennett, T.B.; Cook, N.B.; Nordlund, K.V. Calf respiratory disease and pen microenvironments in naturally ventilated calf barns in winter. *J Dairy Sci*, **2006**; 89(10), pp. 4014-4025.
15. Stanford, K.; McAllister, T.A.; Ayroud, M.; Bray, T.M.; Yost, G.S. Effect of dietary melengestrol acetate on the incidence of acute interstitial pneumonia in feedlot heifers. *Can J Vet Res*, **2006**; 70(3), pp. 218-225.
16. Boyles, S.L.; Loerch, S.C.; Lowe, G.D. Effects of Weaning Management Strategies on Performance and Health of Calves during Feedlot Receiving. *Professional Animal Scientist*, **2007**; 23(6), pp. 637-641.
17. Cusack, P.M.; McMeniman, N.P.; Lean, I.J. Feedlot entry characteristics and climate: their relationship with cattle growth rate, bovine respiratory disease and mortality. *Aust Vet J*, **2007**; 85(8), pp. 311-316.
18. Stevens, E.T.; Thomson, D.U.; Loneragan, G.H.; Lindberg, N. Effects of short-term exposure of feeder cattle to calves persistently infected with bovine viral diarrhea virus. *Bovine Practitioner*, **2007**; 41(2), pp. 151-155.
19. Booker, C.W.; Abutarbush, S.M.; Morley, P.S.; Guichon, P.T.; Wildman, B.K.; Jim, G.K.; Schunicht, O.C.; Pittman, T.J.; Perrett, T.; Ellis, J.A. The effect of bovine viral diarrhea virus infections on health and performance of feedlot cattle. *The Canadian Veterinary Journal*, **2008**; 49(3), pp. 253.
20. Kirkpatrick, J.G.; Step, D.L.; Payton, M.E.; Richards, J.B.; McTague, L.F.; Saliki, J.T.; Confer, A.W.; Cook, B.J.; Ingram, S.H.; Wright, J.C. Effect of age at the time of vaccination on antibody titers and feedlot performance in beef calves. *J Am Vet Med Assoc*, **2008**; 233(1), pp. 136-142.
21. McClary, D.G.; Corbin, M.J.; Carter, B.; Homm, J.; Vogel, G.; Platter, W.; Guthrie, C.A. A comparison of 3-, 5-, 7-, and 10-day post-metaphylaxis evaluation periods on health and performance following on arrival treatment with tilmicosin in feeder cattle - summary of two studies. *Bovine Practitioner*, **2008**; 42, pp. 117-127.
22. Richeson, J.T.; Beck, P.A.; Gadberry, M.S.; Gunter, S.A.; Hess, T.W.; Hubbell, D.S.; rd, J. Effects of on-arrival versus delayed modified live virus vaccination on health, performance, and serum infectious bovine rhinotracheitis titers of newly received beef calves. *J Anim Sci*, **2008**; 86(4), pp. 999-1005.
23. Sanderson, M.W.; Dargatz, D.A.; Wagner, B.A. Risk factors for initial respiratory disease in United States' feedlots based on producer-collected daily morbidity counts. *Can Vet J*, **2008**; 49(4), pp. 373-378.
24. Seeger, J.T.; Grotelueschen, D.M.; Stokka, G.L.; Sides, G.E. Comparison of feedlot health, nutritional performance, carcass characteristics and economic value of unweaned beef calves with an unknown health history and weaned beef calves receiving various herd-of-origin health protocols. *Bovine Practitioner*, **2008**; 42(1), pp. 27-39.
25. Step, D.L.; Krehbiel, C.R.; DePra, H.A.; Cranston, J.J.; Fulton, R.W.; Kirkpatrick, J.G.; Gill, D.R.; Payton, M.E.; Montelongo, M.A.; Confer, A.W. Effects of commingling beef calves from

different sources and weaning protocols during a forty-two-day receiving period on performance and bovine respiratory disease. *J Anim Sci*, **2008**; 86(11), pp. 3146-3158.
26. Gay, E.; Barnouin, J. A nation-wide epidemiological study of acute bovine respiratory disease in France. *Prev. Vet. Med.*, **2009**; 89(3-4), pp. 265-271.
27. Griebel, P.J.; Hodgson, P.D. Abrupt Weaning Significantly Increases Mortality Following a Secondary Bacterial Respiratory Infection. *Proceedings of the 2009 Annual Convention of the American Association of Bovine Practitioners*, **2009**; 42, pp. 23-36.
28. Hessman, B.E.; Fulton, R.W.; Sjeklocha, D.B.; Murphy, T.A.; Ridpath, J.F.; Payton, M.E. Evaluation of economic effects and the health and performance of the general cattle population after exposure to cattle persistently infected with bovine viral diarrhea virus in a starter feedlot. *Am J Vet Res*, **2009**; 70(1), pp. 73-85.
29. Nonnecke, B.J.; Foote, M.R.; Miller, B.L.; Fowler, M.; Johnson, T.E.; Horst, R.L. Effects of chronic environmental cold on growth, health, and select metabolic and immunologic responses of preruminant calves. *Journal of Dairy Science*, **2009**; 92(12), pp. 6134-6143.
30. Richeson, J.T.; Kegley, E.B.; Gadberry, M.S.; Beck, P.A.; Powell, J.G.; Jones, C.A. Effects of on-arrival versus delayed clostridial or modified live respiratory vaccinations on health, performance, bovine viral diarrhea virus type I titers, and stress and immune measures of newly received beef calves. *J Anim Sci*, **2009**; 87(7), pp. 2409-2418.
31. Step, D.L.; Krehbiel, C.R.; Burciaga-Robles, L.O.; Holland, B.R.; Fulton, R.W.; Confer, A.W.; Bechtol, D.T.; Brister, D.L.; Hutcheson, J.R.; Newcomb, H.L. Comparison of single vaccination versus revaccination with a modified-live virus vaccine containing bovine herpesvirus-1, bovine viral diarrhea virus (types la and 2a), parainfluenza type 3 virus, and bovine respiratory syncytial virus in the prevention of bovine respiratory disease in cattle. *Journal of the American Veterinary Medical Association*, **2009**; 235(5), pp. 580-587.
32. Ellis, J.A.; Gow, S.P.; Goji, N. Response to experimentally induced infection with bovine respiratory syncytial virus following intranasal vaccination of seropositive and seronegative calves. *Journal of the American Veterinary Medical Association*, **2010**; 236(9), pp. 991-999.
33. Hanzlicek, G.A.; White, B.J.; Renter, D.G.; Blasi, D.A. A field study evaluating health, performance, and behavior differences in crossbred beef calves administered different vaccine-parasiticide product combinations. *VACCINE*, **2010**; 28(37), pp. 5998-6005.
34. Macek, M.J.; Iliff, J.W.; Olson, K.C.; Jaeger, J.R.; Schmidt, T.B.; Thomson, D.U.; Pacheco, L.A. The relative importance of weaning management and vaccination history on performance by ranch-direct beef calves during weaning and receiving. *Journal of Dairy Science*, **2010**; 93(Suppl. 1), pp. 5.
35. Brscic, M.; Leruste, H.; Heutinck, L.F.; Bokkers, E.A.; Wolthuis-Fillerup, M.; Stockhofe, N.; Gottardo, F.; Lensink, B.J.; Cozzi, G.; Van Reenen, C.G. Prevalence of respiratory disorders in veal calves and potential risk factors. *J Dairy Sci*, **2012**; 95(5), pp. 2753-2764.
36. Cernicchiaro, N.; White, B.J.; Renter, D.G.; Babcock, A.H.; Kelly, L.; Slattery, R. Effects of body weight loss during transit from sale barns to commercial feedlots on health and performance in feeder cattle cohorts arriving to feedlots from 2000 to 2008. *J Anim Sci*, **2012**; 90(6), pp. 1940-1947.
37. Cernicchiaro, N.; White, B.J.; Renter, D.G.; Babcock, A.H.; Kelly, L.; Slattery, R. Associations between the distance traveled from sale barns to commercial feedlots in the United States and overall performance, risk of respiratory disease, and cumulative mortality in feeder cattle during 1997 to 2009. *J Anim Sci*, **2012**; 90(6), pp. 1929-1939.
38. Hodgson, P.D.; Aich, P.; Stookey, J.; Popowych, Y.; Potter, A.; Babiuk, L.; Griebel, P.J. Stress significantly increases mortality following a secondary bacterial respiratory infection. *Vet. Res.*, **2012**; 43, pp. 21.

39. Munson, R.D.; Thomson, D.U.; Reinhardt, C.D. Effects of delayed steroid implanting on health, performance, and carcass quality in high health risk, auction market-sourced feedlot steers. *J Anim Sci*, **2012**; 90(11), pp. 4037-4041.

40. Richeson, J.T.; Kegley, E.B.; Powell, J.G.; Beck, P.A.; Ley, B.L.; Ridpath, J.F. Weaning management of newly received beef calves with or without continuous exposure to a persistently infected bovine viral diarrhea virus pen mate: effects on health, performance, bovine viral diarrhea virus titers, and peripheral blood leukocytes. *J Anim Sci*, **2012**; 90(6), pp. 1972-1985.

41. Wahrmund, J.L.; Burken, D.B.; Wilson, B.K.; Terrill, S.J.; Step, D.L.; Krehbiel, C.R.; Trost, S.M.; Richards, C.J. Effect of truck compartment on ruminal temperature during transit and subsequent health and performance of recently weaned beef heifers. *Professional Animal Scientist*, **2012**; 28(6), pp. 670-677.

42. Richeson, J.T.; Pinedo, P.J.; Kegley, E.B.; Powell, J.G.; Gadberry, M.S.; Beck, P.A.; Falkenberg, S.M. Association of hematologic variables and castration status at the time of arrival at a research facility with the risk of bovine respiratory disease in beef calves. *Journal of the American Veterinary Medical Association*, **2013**; 243(7), pp. 1035-1041.

43. Woolums, A.R.; Berghaus, R.D.; Smith, D.R.; White, B.J.; Engelken, T.J.; Irsik, M.B.; Matlick, D.K.; Lee Jones, A.; Ellis, R.W.; Smith, I.J.; Mason, G.L.; Waggoner, E.R. Producer survey of herd-level risk factors for nursing beef calf respiratory disease. *Journal of the American Veterinary Medical Association*, **2013**; 243(4), pp. 538-547.

44. Griebel, P.; Hill, K.; Stookey, J. How stress alters immune responses during respiratory infection. *Anim Health Res Rev*, **2014**; 15(2), pp. 161-165.

45. Hay, K.E.; Barnes, T.S.; Morton, J.M.; Clements, A.C.A.; Mahony, T.J. *Risk factors for bovine respiratory disease in Australian feedlot cattle: using a causal diagram-informed approach to estimate total effects of mixing and group size before feedlot entry*, **2014**; Society for Veterinary Epidemiology and Preventive Medicine: Dublin. p. 99-109.

46. Hay, K.E.; Barnes, T.S.; Morton, J.M.; Clements, A.C.; Mahony, T.J. Risk factors for bovine respiratory disease in Australian feedlot cattle: use of a causal diagram-informed approach to estimate effects of animal mixing and movements before feedlot entry. *Prev Vet Med*, **2014**; 117(1), pp. 160-169.

47. Williams, D.R.; Pithua, P.; Garcia, A.; Champagne, J.; Haines, D.M.; Aly, S.S. Effect of Three Colostrum Diets on Passive Transfer of Immunity and Preweaning Health in Calves on a California Dairy following Colostrum Management Training. *Vet Med Int*, **2014**; 2014, pp. 698741.

48. Bailey, E.A.; Jaeger, J.R.; Schmidt, T.B.; Waggoner, J.W.; Pacheco, L.A.; Thomson, D.U.; Olson, K.C. Effects of weaning period length on growth and health of preconditioned, spring-born beef calves originating from the Great Plains. I. Conventional weaning ages. *Professional Animal Scientist*, **2015**; 31(1), pp. 20-29.

49. Bailey, E.A.; Jaeger, J.R.; Schmidt, T.B.; Waggoner, J.W.; Pacheco, L.A.; Thomson, D.U.; Olson, K.C. Effects of weaning-period length on growth and health of preconditioned, spring-born beef calves originating from the Great Plains. II. Early weaning. *Professional Animal Scientist*, **2015**; 31(1), pp. 30-39.

50. Richeson, J.T.; Beck, P.A.; Poe, K.D.; Gadberry, M.S.; Hess, T.W.; Hubbell, D.S. Effects of administration of a modified-live virus respiratory vaccine and timing of vaccination on health and performance of high-risk beef stocker calves. *Bovine Practitioner*, **2015**; 49(1), pp. 37-42.

51. Bailey, E.A.; Jaeger, J.R.; Schmidt, T.B.; Waggoner, J.W.; Pacheco, L.A.; Thomson, D.U.; Olson, K.C. Effects of number of viral respiratory disease vaccinations during preconditioning on

health, performance, and carcass merit of ranch-direct beef calves during receiving and finishing. *Professional Animal Scientist*, **2016**; 32(3), pp. 271-278.

52. Bras, A.L.; Barkema, H.W.; Woodbury, M.; Ribble, C.; Perez-Casal, J.; Windeyer, M.C. Risk factors for Mycoplasma bovis-associated disease in farmed bison (Bison bison) herds in western Canada: A case-control study. *Prev Vet Med*, **2016**; 129, pp. 67-73.
53. Hay, K.E.; Morton, J.M.; Clements, A.C.; Mahony, T.J.; Barnes, T.S. Associations between feedlot management practices and bovine respiratory disease in Australian feedlot cattle. *Prev Vet Med*, **2016**; 128, pp. 23-32.
54. Hay, K.E.; Morton, J.M.; Schibrowski, M.L.; Clements, A.C.; Mahony, T.J.; Barnes, T.S. Associations between prior management of cattle and risk of bovine respiratory disease in feedlot cattle. *Prev Vet Med*, **2016**; 127, pp. 37-43.
55. Lippolis, K.D.; **Cooke**, R.F.; Schubach, K.M.; Brandão, A.P.; Silva, L.G.; Marques, R.S.; Bohnert, D.W. Altering the time of vaccination against respiratory pathogens to enhance antibody response and performance of feeder cattle. *J Anim Sci*, **2016**; 94(9), pp. 3987-3995.
56. Murray, G.M.; Cassidy, J.P.; Clegg, T.A.; Tratalos, J.A.; McClure, J.; O'Neill, R.G.; Sammin, D.J.; Casey, M.J.; McElroy, M.; Earley, B.; Bourke, N.; More, S.J. A retrospective epidemiological analysis of risk factors for a primary necropsy diagnosis of bovine respiratory disease. Prev Vet Med, **2016**; 132, pp. 49-56.
57. Rogers, K.C.; Miles, D.G.; Renter, D.G.; Sears, J.E.; Woodruff, J.L. Effects of delayed respiratory viral vaccine and/or inclusion of an immunostimulant on feedlot health, performance, and carcass merits of auction-market derived feeder heifers. Bovine Practitioner, **2016**; 50(2), pp. 154-162.
58. Hay, K.E.; Morton, J.M.; Clements, A.C.A.; Mahony, T.J.; Barnes, T.S. Population-level effects of risk factors for bovine respiratory disease in Australian feedlot cattle. Prev Vet Med, **2017**; 140, pp. 78-86.
59. Pempek, J.; Trearchis, D.; Masterson, M.; Habing, G.; Proudfoot, K. Veal calf health on the day of arrival at growers in Ohio. J Anim Sci, **2017**; 95(9), pp. 3863-3872.
60. Griffin, C.M.; Scott, J.A.; Karisch, B.B.; Woolums, A.R.; Blanton, J.R.; Kaplan, R.M.; Epperson, W.B.; Smith, D.R. A randomized controlled trial to test the effect of on-arrival vaccination and deworming on stocker cattle health and growth performance. Bov Pract (Stillwater), **2018**; 52(1), pp. 26-33.
61. Lopez, F.A.; Oosthuysen, E.R.; Duff, G.C.; Richeson, J.T.; Samuelson, K.L.; Hubbert, M.E.; Löest, C.A. Health, performance, and complete blood counts of newly received feedlot heifers in response to an oral drench of water and crude glycerin. Translational Animal Science, **2018**; 2, pp. S74-S78.
62. Louie, A.P.; Rowe, J.D.; Love, W.J.; Lehenbauer, T.W.; Aly, S.S. Effect of the environment on the risk of respiratory disease in preweaning dairy calves during summer months. Journal of Dairy Science, **2018**; 101(11), pp. 10230-10247.
63. Perrett, T.; Johnson, D.L.; Song, J.; van de Pol, S.; Dahlman, D.A.; Rademacher, R.D.; Hannon, S.J.; Booker, C.W. A retrospective analysis of feedlot morbidity and mortality outcomes in calves born to dams with known viral vaccination history. Can Vet J, **2018**; 59(7), pp. 779-782.
64. Schibrowski, M.L.; Gibson, J.S.; Hay, K.E.; Mahony, T.J.; Barnes, T.S. Mycoplasma bovis and bovine respiratory disease: A risk factor study in Australian feeder cattle. Prev Vet Med, **2018**; 157, pp. 152-161.
65. Shane, D.D.; McLellan, J.G.; White, B.J.; Larson, R.L.; Amrine, D.E.; Sanderson, M.W.; Apley, M.D. Evaluation of animal-to-animal and community contact structures determined by a real-time location system for correlation with and prediction of new bovine respiratory

disease diagnoses in beef cattle during the first 28 days after feedlot entry. Am J Vet Res, **2018**; 79(12), pp. 1277-1286.
66. Woolsoncroft, M.A.; Youngers, M.E.; McPhillips, L.J.; Lockard, C.G.; Haviland, C.L.; DeSocio, E.S.; Ryan, W.R.; Richards, C.J.; Wilson, B.K. Effects of exercise and roughage source on the health and performance of receiving beef calves. Professional Animal Scientist, **2018**; 34(2), pp. 183-191.
67. Dewell, R.D.; Millman, S.T.; Parsons, R.L.; Sadler, L.J.; Noffsinger, T.H.; Busby, W.D.; Wang, C.; Dewell, G.A. Clinical trial to assess the impact of acclimation and low-stress cattle handling on bovine respiratory disease and performance during the feedyard finishing phase. Bovine Practitioner, **2019**; 53(1), pp. 71-80.
68. Schubach, K.; Cooke, R.F.; Brandao, A.; Schumaher, T.; Souza, O.; Bohnert, D.; Marques, R. Altering the time of vaccination against respiratory pathogens enhanced antibody response and health of feedlot cattle. Journal of Animal Science, **2019**; 97(Suppl. 1), pp. 39-40.
69. Schumaher, T.F.; Cooke, R.F.; Brandão, A.P.; Schubach, K.M.; e Sousa, O.A.; Bohnert, D.W.; Marques, R.S. Effects of vaccination timing against respiratory pathogens on performance, antibody response, and health in feedlot cattle. J Anim Sci, **2019**; 97(2), pp. 620-630.
70. Tomczak, D.J.; Samuelson, K.L.; Jennings, J.S.; Richeson, J.T. Oral hydration therapy with water affects health and performance of high-risk, newly received feedlot cattle. Applied Animal Science, **2019**; 35(1), pp. 30-38.
71. Taylor, J.D.; Gilliam, J.N.; Mourer, G.; Stansberry, C. Comparison of effects of four weaning methods on health and performance of beef calves. Animal, **2020**; 14(1), pp. 161-170.
72. Wilson, D.J.; Stojkov, J.; Renaud, D.L.; Fraser, D. Risk factors for poor health outcomes for male dairy calves undergoing transportation in western Canada. CAN. VET. J., **2020**; 61(12), pp. 1265-1272.
73. van Leenen, K.; Jouret, J.; Demeyer, P.; Vermeir, P.; Leenknecht, D.; Van Driessche, L.; De Cremer, L.; Masmeijer, C.; Boyen, F.; Deprez, P.; Cox, E.; Devriendt, B.; Pardon, B. Particulate matter and airborne endotoxin concentration in calf barns and their association with lung consolidation, inflammation, and infection. *Journal of Dairy Science*, **2021**; 104(5), pp. 5932-5947.

Metaphylaxis:

1. Schumann, F.J.; Janzen, E.D.; McKinnon, J.J. Prophylactic tilmicosin medication of feedlot calves at arrival. *CAN. VET. J.*, **1990**; 31(4), pp. 285-288.
2. Harland, R.J.; Jim, G.K.; Guichon, P.T.; Townsend, H.G.; Janzen, E.D. Efficacy of parenteral antibiotics for disease prophylaxis in feedlot calves. *Can Vet J*, **1991**; 32(3), pp. 163-168.
3. Schumann, F.J.; Janzen, E.D.; McKinnon, J.J. Prophylactic medication of feedlot calves with tilmicosin. *Vet Rec*, **1991**; 128(12), pp. 278-280.
4. Guichon, P.T.; Booker, C.W.; Jim, G.K. Comparison of two formulations of oxytetracycline given prophylactically to reduce the incidence of bovine respiratory disease in feedlot calves. *Can Vet J*, **1993**; 34(12), pp. 736-741.
5. Morck, D.W.; Merrill, J.K.; Thorlakson, B.E.; Olson, M.E.; Tonkinson, L.V.; Costerton, J.W. Prophylactic efficacy of tilmicosin for bovine respiratory tract disease. *J Am Vet Med Assoc*, **1993**; 202(2), pp. 273-277.
6. Merrill, J.K.; Jim, G.K.; Guichon, P.T.; Booker, C.W. A comparison of the prophylactic use of tilmicosin injection and long-acting oxytetracycline injection on morbidity, mortality, and performance of feedlot calves. *J Anim Sci*, **1994**; 72(SUPPL. 1), pp. 143.

7. Van Donkersgoed, J.; Janzen, E.D.; Potter, A.A.; Harland, R.J. The occurrence of Haemophilus somnus in feedlot calves and its control by postarrival prophylactic mass medication. *Can Vet J*, **1994**; 35(9), pp. 573-580.
8. Gallo, G.F.; Berg, J.L. Efficacy of a feed-additive antibacterial combination for improving feedlot cattle performance and health. *Can Vet J*, **1995**; 36(4), pp. 223-229.
9. Galyean, M.L.; Gunter, S.A.; Malcolm-Callis, K.J. Effects of arrival medication with tilmicosin phosphate on health and performance of newly received beef cattle. *J Anim Sci*, **1995**; 73(5), pp. 1219-1226.
10. Scott, P.R. Efficacy of strategic tilmicosin injection during an outbreak of respiratory disease in housed beef calves. *Br. Vet. J.*, **1995**; 151(5), pp. 587-589.
11. Hughes, T.A.; Tice, G.A.; O'Connor, D. An evaluation of the use of Micotil, metaphylactically, on arrival, for calf pneumonia. *Ir. Vet. J.*, **1996**; 49(10), pp. 622-624.
12. Vogel, G.J.; Laudert, S.B.; Zimmermann, A.; Guthrie, C.A.; Mechor, G.D.; Moore, G.M. Effects of tilmicosin on acute undifferentiated respiratory tract disease in newly arrived feedlot cattle. *J Am Vet Med Assoc*, **1998**; 212(12), pp. 1919-1924.
13. McClary, D.; Vogel, G. Effect of timing of tilmicosin metaphylaxis on control of bovine respiratory disease and performance in feeder cattle. *Bovine Practitioner*, **1999**; 33(2), pp. 155-161.
14. Duff, G.C.; Walker, D.A.; Malcolm-Callis, K.J.; Wiseman, M.W.; Hallford, D.M. Effects of preshipping vs. arrival medication with tilmicosin phosphate and feeding chlortetracycline on health and performance of newly received beef cattle. *J Anim Sci*, **2000**; 78(2), pp. 267-274.
15. Frank, G.H.; Duff, G.C. Effects of tilmicosin phosphate, administered prior to transport or at time of arrival, and feeding of chlortetracycline, after arrival in a feedlot, on Mannheimia haemolytica in nasal secretions of transported steers. *Am J Vet Res*, **2000**; 61(12), pp. 1479-1483.
16. Frank, G.H.; Briggs, R.E.; Duff, G.C.; Loan, R.W.; Purdy, C.W. Effects of vaccination prior to transit and administration of florfenicol at time of arrival in a feedlot on the health of transported calves and detection of Mannheimia haemolytica in nasal secretions. *Am J Vet Res*, **2002**; 63(2), pp. 251-256.
17. Schunicht, O.C.; Guichon, P.T.; Booker, C.W.; Jim, G.K.; Wildman, B.K.; Hill, B.W.; Ward, T.I.; Bauck, S.W.; Jacobsen, J.A. A comparison of prophylactic efficacy of tilmicosin and a new formulation of oxytetracycline in feedlot calves. *Can Vet J*, **2002**; 43(5), pp. 355-362.
18. Guthrie, C.A.; Rogers, K.C.; Christmas, R.A.; Vogel, G.J.; Laudert, S.B.; Mechor, G.D. Efficacy of metaphylactic tilmicosin for controlling bovine respiratory disease in high-risk northern feeder calves. *Bovine Practitioner*, **2004**; 38(1), pp. 46-53.
19. Godinho, K.S.; Wolf, R.M.L.G.; Sherington, J.; Rowan, T.G.; Sunderland, S.J.; Evans, N.A. Efficacy of tulathromycin in the treatment and prevention of natural outbreaks of bovine respiratory disease in European cattle. *Vet. Ther.*, **2005**; 6(2), pp. 122-135.
20. Kilgore, W.R.; Spensley, M.S.; Sun, F.; Nutsch, R.G.; Rooney, K.A.; Skogerboe, T.L. Clinical effectiveness of tulathromycin, a novel triamilide antimicrobial, for the control of respiratory disease in cattle at high risk for developing bovine respiratory disease. *Vet Ther*, **2005**; 6(2), pp. 136-142.
21. Rooney, K.A.; Nutsch, R.G.; Skogerboe, T.L.; Weigel, D.J.; Gajewski, K.; Kilgore, W.R. Efficacy of tulathromycin compared with tilmicosin and florfenicol for the control of respiratory disease in cattle at high risk of developing bovine respiratory disease. *Vet Ther*, **2005**; 6(2), pp. 154-166.

22. Booker, C.W.; Schunicht, O.C.; Guichon, P.T.; Jim, G.K.; Wildman, B.K.; Pittman, T.J.; Perrett, T. An evaluation of the metaphylactic effect of ceftiofur crystalline free Acid in feedlot calves. *Vet Ther*, **2006**; 7(3), pp. 257-274.
23. Booker, C.W.; Abutarbush, S.M.; Schunicht, O.C.; Jim, G.K.; Perrett, T.; Wildman, B.K.; Guichon, P.T.; Pittman, T.J.; Jones, C.; Pollock, C.M. Evaluation of the efficacy of tulathromycin as a metaphylactic antimicrobial in feedlot calves. *Vet Ther*, **2007**; 8(3), pp. 183-200.
24. Martín, G.J.V.; Partida, E.L.; Villalobos, P.N.; López, C.M.; López-Guerrero, C.E.; Blanco, A.S. Evaluation of mass and selective metaphylaxis medication with florfenicol at feedlot entry as a tool against bovine respiratory disease under commercial conditions in Spain. *Cattle Practice*, **2007**; 15(3), pp. 309-311.
25. Step, D.L.; Engelken, T.; Romano, C.; Holland, B.; Krehbiel, C.; Johnson, J.C.; Bryson, W.L.; Tucker, C.M.; Robb, E.J. Evaluation of three antimicrobial regimens used as metaphylaxis in stocker calves at high risk of developing bovine respiratory disease. *Vet. Ther.*, **2007**; 8(2), pp. 136-147.
26. Catry, B.; Duchateau, L.; Van De Ven, J.; Laevens, H.; Opsomer, G.; Haesebrouck, F.; De Kruif, A. Efficacy of metaphylactic florfenicol therapy during natural outbreaks of bovine respiratory disease. *J. Vet. Pharmacol. Ther.*, **2008**; 31(5), pp. 479-487.
27. Cusack, P.M. Effects of a dietary complex of humic and fulvic acids (FeedMAX 15) on the health and production of feedlot cattle destined for the Australian domestic market. *Aust Vet J*, **2008**; 86(1-2), pp. 46-49.
28. Johnson, J.C.; Bryson, W.L.; Barringer, S.; Hunsaker, B.D. Evaluation of on-arrival versus prompted metaphylaxis regimes using ceftiofur crystalline free acid for feedlot heifers at risk of developing bovine respiratory disease. *Vet Ther*, **2008**; 9(1), pp. 53-62.
29. McClary, D.G.; Corbin, M.J.; Carter, B.; Homm, J.; Vogel, G.; Platter, W.; Guthrie, C.A. A comparison of 3-, 5-, 7-, and 10-day post-metaphylaxis evaluation periods on health and performance following on arrival treatment with tilmicosin in feeder cattle - summary of two studies. *Bovine Practitioner*, **2008**; 42, pp. 117-127.
30. Montgomery, S.P.; Sindt, J.J.; Greenquist, M.A.; Loe, E.R.; Drouillard, J.S. Comparison of bovine transfer factor and tilmicosin phosphate: effects on health and growth performance of newly arrived feedlot heifers. *International Journal of Applied Research in Veterinary Medicine*, **2008**; 6(3), pp. 175-180.
31. Nickell, J.S.; White, B.J.; Larson, R.L.; Blasi, D.A.; Renter, D.G. Comparison of short-term health and performance effects related to prophylactic administration of tulathromycin versus tilmicosin in long-hauled, highly stressed beef stocker calves. *Vet. Ther.*, **2008**; 9(2), pp. 147-156.
32. Van Donkersgoed, J.; Merrill, J.; Hendrick, S. Comparative efficacy of tilmicosin versus tulathromycin as a metaphylactic antimicrobial in feedlot calves at moderate risk for respiratory disease. *Vet Ther*, **2008**; 9(4), pp. 291-297.
33. Corbin, M.J.; Gould, J.A.; Carter, B.L.; McClary, D.G.; Portillo, T.A. Effects and Economic Implications of Metaphylactic Treatment of Feeder Cattle with Two Different Dosages of Tilmicosin on the Incidence of Bovine Respiratory Disease (BRD) - A Summary of Two Studies. *Bovine Practitioner*, **2009**; 43(2), pp. 140-152.
34. Wallace, J.O.; Reinhardt, C.D.; Thomson, D.U. Effects of Concurrent Metaphylaxis with Chlortetracycline and Tulathromycin on the Health and Performance of High-risk Beef Calves. *Bovine Practitioner*, **2009**; 43(1), pp. 14-17.

35. Rossi, S.C.A.; Vandoni, S.L.; Bonfanti, M.; Forbes, A.B. Effects of Arrival Medication with Gamithromycin on Bovine Respiratory Disease in Feedlot Cattle in Italy. *International Journal of Applied Research in Veterinary Medicine*, **2010**; 8(2), pp. 87-96.
36. Stanton, A.L.; Kelton, D.F.; LeBlanc, S.J.; Millman, S.T.; Wormuth, J.; Dingwell, R.T.; Leslie, K.E. The effect of treatment with long-acting antibiotic at postweaning movement on respiratory disease and on growth in commercial dairy calves. *Journal of Dairy Science*, **2010**; 93(2), pp. 574-581.
37. Gonzalez-Martin, J.V.; Elvira, L.; Cervino Lopez, M.; Perez Villalobos, N.; Calvo Lopez-Guerrero, E.; Astiz, S. Reducing antibiotic use: Selective metaphylaxis with florfenicol in commercial feedlots. *Livestock Science*, **2011**; 141(2-3), pp. 173-181.
38. Baggott, D.; Casartelli, A.; Fraisse, F.; Manavella, C.; Marteau, R.; Rehbein, S.; Wiedemann, M.; Yoon, S. Demonstration of the metaphylactic use of gamithromycin against bacterial pathogens associated with bovine respiratory disease in a multicentre farm trial. *Vet. Rec.*, **2011**; 168(9), pp. 241.
39. Lechtenberg, K.; Daniels, C.S.; Royer, G.C.; Bechtol, D.T.; Chester, S.T.; Blair, J.; Tessman, R.K. Field Efficacy Study of Gamithromycin for the Control of Bovine Respiratory Disease in Cattle at High Risk of Developing the Disease. *International Journal of Applied Research in Veterinary Medicine*, **2011**; 9(2), pp. 184-192.
40. Lechtenberg, K.; Tessman, R.K.; Romano, D. Efficacy of Gamithromycin Injectable Solution for Control of Pneumonia in Cattle Challenged with Histophilus somni after Treatment. *International Journal of Applied Research in Veterinary Medicine*, **2011**; 9(3), pp. 241-248.
41. Van Donkersgoed, J.; Merrill, J.K. A comparison of tilmicosin to gamithromycin for on arrival treatment of bovine respiratory disease in feeder steers. *Bovine Practitioner*, **2012**; 46(1), pp. 46-51.
42. Hendrick, S.H.; Bateman, K.G.; Rosengren, L.B. The effect of antimicrobial treatment and preventive strategies on bovine respiratory disease and genetic relatedness and antimicrobial resistance of Mycoplasma bovis isolates in a western Canadian feedlot. *Can Vet J*, **2013**; 54(12), pp. 1146-1156.
43. Stegner, J.E.; Lucas, M.J.; McLaughlin, C.L.; Davis, M.S.; Alaniz, G.R.; Weigel, D.J.; Pollreisz, J.H.; Tucker, C.M.; Koers, W.C.; Turgeon, O.A.; Szasz, J.I. Comparative effects of therapeutic programs on bovine respiratory disease, performance, carcass, and profitability of high-risk feedlot heifers. *Professional Animal Scientist*, **2013**; 29(3), pp. 208-218.
44. Torres, S.; Thomson, D.U.; Bello, N.M.; Nosky, B.J.; Reinhardt, C.D. Field study of the comparative efficacy of gamithromycin and tulathromycin for the control of undifferentiated bovine respiratory disease complex in beef feedlot calves at high risk of developing respiratory tract disease. *Am J Vet Res*, **2013**; 74(6), pp. 839-846.
45. Van Donkersgoed, J.; Merrill, J.K. Efficacy of tilmicosin for on-arrival treatment of bovine respiratory disease in backgrounded winter-placed feedlot calves. *Bovine Practitioner*, **2013**; 47(1), pp. 7-12.
46. Van Donkersgoed, J.; Merrill, J.K. Efficacy of tilmicosin and tildipirosin for on-arrival treatment of bovine respiratory disease in fall-placed feedlot calves in western Canada. *Bovine Practitioner*, **2013**; 47(2), pp. 146-151.
47. Amrine, D.; White, B.; Goehl, D.; Sweiger, S.H.; Nosky, B.; Tessman, R.K. Comparisons of Metaphylactic Treatments of Zactran (R) (gamithromycin) vs. Excede (R) (ceftiofur crystalline free acid) in High Risk, Stocker Calves. *International Journal of Applied Research in Veterinary Medicine*, **2014**; 12(3), pp. 221-228.

48. Thomson, D.U.; Swingle, R.S.; Branine, M.; Bartle, S.J.; Yates, D.A. Effects of timing of chlortetracycline in combination with decoquinate on growth performance, health and carcass characteristics of feeder steers. *Bov. Pract.*, **2014**; 48, pp. 120-128.
49. Fazzio, L.E.; Giuliodori, M.J.; Galván, W.R.; Streitenberger, N.; Landoni, M.F. A metaphylactic treatment with double dose oxytetracycline reduces the risk of bovine respiratory disease in feedlot calves. *Revista Veterinaria*, **2015**; 26(2), pp. 89-92.
50. Stanford, K.; Gibb, D.J.; Schwartzkopf-Genswein, K.S.; van Herk, F.; McAllister, T.A. Feeding subtherapeutic antimicrobials to low-risk cattle does not confer consistent performance benefits. *Canadian Journal of Animal Science*, **2015**; 95(4), pp. 589-597.
51. Confer, A.W.; Snider, T.A.; Taylor, J.D.; Montelongo, M.; Sorensen, N.J. Clinical disease and lung lesions in calves experimentally inoculated with histophilus somni five days after metaphylactic administration of tildipirosin or tulathromycin. *American Journal of Veterinary Research*, **2016**; 77(4), pp. 358-366.
52. Crepieux, T.; Miller, C.; Regev-Shoshani, G.; Schaefer, A.; Dorin, C.; Alexander, T.; Timsit, E. Randomized, non-inferiority trial comparing a nitric oxide releasing solution with a macrolide antibiotic for control of bovine respiratory disease in beef feedlot calves at high-risk of developing respiratory tract disease. *Res Vet Sci*, **2016**; 105, pp. 216-221.
53. Hanzlicek, G.A.; Blasi, D.A.; Oleen, B.E.; Anderson, G.A. A randomized field study comparing differences in core body temperature, health, and performance in crossbred beef heifers administered 2 antimicrobial products given upon arrival at a stocker facility. *Professional Animal Scientist*, **2016**; 32(4), pp. 438-444.
54. Miller, T.J.; Hubbert, M.E.; Reinhardt, C.D.; Löest, C.A.; Schwandt, E.F.; Thomson, D.U. Comparison of tulathromycin, tilmicosin, and gamithromycin for metaphylactic treatment of high-risk calves for control of bovine respiratory disease. *Bovine Practitioner*, **2016**; 50(2), pp. 175-179.
55. Hill, T.M.; Quigley, J.D.; Suarez-Mena, F.X.; Dennis, T.S.; Schlotterbeck, R.L. Case Study: Control of bovine respiratory disease in dairy calves with tulathromycin and effect on calf health and performance from 0 to 4 months of age. *Prof Anim Sci*, **2017**; 33(4), pp. 498-503.
56. Magalhaes, L.Q.; Baptista, A.L.; Fonseca, P.d.A.; Menezes, G.L.; Nogueira, G.M.; Headley, S.A.; Tomazi Fritzen, J.T.; Alfieri, A.A.; Elsen Saut, J.P. Use of metaphylactic protocols based on the risk to develop bovine respiratory diseases in feedlot cattle. *Ciencia Rural*, **2017**; 47(8), pp. e20161110.
57. Sturgess, K.L.; Renter, D.G. Comparison of tulathromycin, tildipirosin, and tilmicosin for control of bovine respiratory disease in steers purchased from auction markets and fed in a Texas feedlot. *Bovine Practitioner*, **2017**; 51(1), pp. 17-24.
58. Timsit, E.; Workentine, M.; Crepieux, T.; Miller, C.; Regev-Shoshani, G.; Schaefer, A.; Alexander, T. Effects of nasal instillation of a nitric oxide-releasing solution or parenteral administration of tilmicosin on the nasopharyngeal microbiota of beef feedlot cattle at high-risk of developing respiratory tract disease. *Res Vet Sci*, **2017**; 115, pp. 117-124.
59. Van Donkersgoed, J.; Hendrick, S.; Nickel, T. Comparison of gamithromycin and tildipirosin for metaphylaxis treatment of winter-placed feedlot calves for control of bovine respiratory disease. *Bovine Practitioner*, **2017**; 51(2), pp. 184-189.
60. Rivera, J.D.; Johnson, J.T.; Blue, G.K. Effects of oral tilmicosin on health and performance in newly received beef heifers. *Professional Animal Scientist*, **2018**; 34(1), pp. 42-50.
61. Szasz, J.I.; McMurphy, C.P.; Bryant, T.C.; Luque, J.; Barcelo, C.; Sepulveda, G.; Blood, K.S.; Bernhard, B.C.; Hughes, H.D. Influence of therapeutic use of feedgrade tetracyclines in combination with tulathromycin metaphylaxis on animal health and performance of Holstein steer calves. *Translational Animal Science*, **2019**; 3(1), pp. 185-194.

62. Valencia, D.M.A.; Liebstein, M.L.; Thompson, P.A.R.; Renter, D.G. A randomized trial comparing effects of respiratory disease metaphylaxis with gamithromycin or ceftiofur crystalline free acid on the health, performance, and economic return of auction market-derived stocker calves backgrounded on Missouri pastures. *Bovine Practitioner*, **2019**; 53(1), pp. 10-17.
63. Munoz, V.I.; Samuelson, K.L.; Tomczak, D.J.; Seiver, H.A.; Smock, T.M.; Richeson, J.T. Comparative efficacy of metaphylaxis with tulathromycin and pentavalent modified-live virus vaccination in high-risk, newly received feedlot cattle. *Applied Animal Science*, **2020**; 36(6), pp. 799-807.
64. Word, A.B.; Ellis, G.B.; Holland, B.P.; Streeter, M.N.; Hutcheson, J.P. Effects of antimicrobial metaphylaxis using no antimicrobial, tilmicosin, or tildipirosin and 2 different days on feed on the health and growth performance of lightweight beef steer calves originating from Mexico. *Applied Animal Science*, **2021**; 37(2), pp. 207-216.

Vaccination:

1. Thorlakson, B.; Martin, W.; Peters, D. A field trial to evaluate the efficacy of a commercial Pasteurella haemolytica bacterial extract in preventing bovine respiratory disease. *Can Vet J*, **1990**; 31(8), pp. 573-579.
2. Van Donkersgoed, J.; Janzen, E.D.; Townsend, H.G.; Durham, P.J. Five field trials on the efficacy of a bovine respiratory syncytial virus vaccine. *Can Vet J*, **1990**; 31(2), pp. 93-100.
3. Harland, R.J.; Potter, A.A.; van Drunen-Littel-van den Hurk, S.; Van Donkersgoed, J.; Parker, M.D.; Zamb, T.J.; Janzen, E.D. The effect of subunit or modified live bovine herpesvirus-1 vaccines on the efficacy of a recombinant Pasteurella haemolytica vaccine for the prevention of respiratory disease in feedlot calves. *Can Vet J*, **1992**; 33(11), pp. 734-741.
4. Hill, W.J.; Kirkpatrick, J.; Gill, D.R.; Ball, R.L. The effects of septimune on health and performance of stressed stocker cattle. *Animal Science Research Report, Agricultural Experiment Station, Oklahoma State University*, **1993**(No. P-933), pp. 301-303.
5. Van Donkersgoed, J.; Schumann, F.J.; Harland, R.J.; Potter, A.A.; Janzen, E.D. The effect of route and dosage of immunization on the serological response to a Pasteurella haemolytica and Haemophilus somnus vaccine in feedlot calves. *Can Vet J*, **1993**; 34(12), pp. 731-735.
6. Van Drunen Littel-Van Den Hurk, S.; Van Donkersgoed, J.; Kowalski, J.; Van Den Hurki, J.V.; Harland, R.; Babiuk, L.A.; Zamb, T.J. A subunit gIV vaccine, produced by transfected mammalian cells in culture, induces mucosal immunity against bovine herpesvirus-1 in cattle. *VACCINE*, **1994**; 12(14), pp. 1295-1302.
7. Kennedy, J.A. The effects of Re-17 mutant Salmonella typhimurium bacterin-toxoid on bovine respiratory disease in feedlot heifers. *Agri-Practice*, **1995**; 16(3), pp. 29-31.
8. Morton, R.J.; Panciera, R.J.; Fulton, R.W.; Frank, G.H.; Ewing, S.A.; Homer, J.T.; Confer, A.W. Vaccination of cattle with outer membrane protein-enriched fractions of Pasteurella haemolytica and resistance against experimental challenge exposure. *American Journal of Veterinary Research*, **1995**; 56(7), pp. 875-879.
9. Rice Conlon, J.A.; Gallo, G.F.; Shewen, P.E.; Adlam, C. Comparison of protection of experimentally challenged cattle vaccinated once or twice with a Pasteurella haemolytica bacterial extract vaccine. *CAN. J. VET. RES.*, **1995**; 59(3), pp. 179-182.
10. Bosch, J.C.; Kaashoek, M.J.; Kroese, A.H.; Van Oirschot, J.T. An attenuated bovine herpesvirus 1 marker vaccine induces a better protection than two inactivated marker vaccines. *VET. MICROBIOL.*, **1996**; 52(3-4), pp. 223-234.

11. Sreevatsan, S.; Ames, T.R.; Werdin, R.E.; Yoo, H.S.; Maheswaran, S.K. Evaluation of three experimental subunit vaccines against pneumonic pasteurellosis in cattle. *VACCINE*, **1996**; 14(2), pp. 147-154.
12. Haanes, E.J.; Guimond, P.; Wardley, R. The bovine parainfluenza virus type-3 (BPIV-3) hemagglutinin/neuraminidase glycoprotein expressed in baculovirus protects calves against experimental BPIV-3 challenge. *VACCINE*, **1997**; 15(6-7), pp. 730-738.
13. Van Drunen Littel-Van Den Hurk, S.; Tikoo, S.K.; Van Den Hurk, J.V.; Babiuk, L.A.; Van Donkersgoed, J. Protective immunity in cattle following vaccination with conventional and marker bovine herpesvirus-1 (BHV1) vaccines. *VACCINE*, **1997**; 15(1), pp. 36-44.
14. Mosier, D.A.; Panciera, R.J.; Rogers, D.P.; Uhlich, G.A.; Butine, M.D.; Confer, A.W.; Basaraba, R.J. Comparison of serologic and protective responses induced by two Pasteurella vaccines. *Can J Vet Res*, **1998**; 62(3), pp. 178-182.
15. Bryson, D.G.; Adair, B.M.; McNulty, M.S.; McAliskey, M.; Bradford, H.E.L.; Allan, G.M.; Evans, R.T.; Forster, F. Studies on the efficacy of intranasal vaccination for the prevention of experimentally induced parainfluenza type 3 virus pneumonia in calves. *Vet. Rec.*, **1999**; 145(2), pp. 33-39.
16. Bingham, H.R.; Morley, P.S.; Wittum, T.E.; Bray, T.M.; Ellis, J.A.; Queen, W.G.; Shulaw, W.P. Effects of 3-methylindole production and immunity against bovine respiratory syncytial virus on development of respiratory tract disease and rate of gain of feedlot cattle. *Am J Vet Res*, **2000**; 61(10), pp. 1309-1314.
17. Duff, G.C.; Malcolm-Callis, K.J.; Walker, D.A.; Wiseman, M.W.; Galyean, M.L.; Perino, L.J. Effects of intranasal versus intramuscular modified live vaccines and vaccine timing on health and performance by newly received beef cattle. *Bov. Pract.*, **2000**; 34(1), pp. 66-71.
18. Gummow, B.; Mapham, P.H. A stochastic partial-budget analysis of an experimental Pasteurella haemolytica feedlot vaccine trial. *Prev Vet Med*, **2000**; 43(1), pp. 29-42.
19. Confer, A.W.; Ayalew, S.; Panciera, R.J.; Montelongo, M.; Whitworth, L.C.; Hammer, J.D. Immunogenicity of recombinant Mannheimia haemolytica serotype 1 outer membrane protein PlpE and augmentation of a commercial vaccine. *VACCINE*, **2003**; 21(21-22), pp. 2821-2829.
20. Kerkhofs, P.; Renjifo, X.; Toussaint, J.F.; Letellier, C.; Vanopdenbosch, E.; Wellemans, G. Enhancement of the immune response and virological protection of calves against bovine herpesvirus type 1 with an inactivated gE-deleted vaccine. *Vet. Rec.*, **2003**; 152(22), pp. 681-686.
21. MacGregor, S.; Smith, D.; Perino, L.J.; Hunsaker, B.D. An evaluation of the effectiveness of a commercial Mannheimia (Pasteurella) haemolytica vaccine in a commercial feedlot. *Bovine Practitioner*, **2003**; 37(1), pp. 78-82.
22. Schunicht, O.C.; Booker, C.W.; Jim, G.K.; Guichon, P.T.; Wildman, B.K.; Hill, B.W. Comparison of a multivalent viral vaccine program versus a univalent viral vaccine program on animal health, feedlot performance, and carcass characteristics of feedlot calves. *Can Vet J*, **2003**; 44(1), pp. 43-50.
23. Woolums, A.R.; Siger, L.; Johnson, S.; Gallo, G.; Conlon, J. Rapid onset of protection following vaccination of calves with multivalent vaccines containing modified-live or modified-live and killed BHV-1 is associated with virus-specific interferon gamma production. *VACCINE*, **2003**; 21(11-12), pp. 1158-1164.
24. Fairbanks, K.F.; Campbell, J.; Chase, C.C. Rapid onset of protection against infectious bovine rhinotracheitis with a modified-live virus multivalent vaccine. *Vet Ther*, **2004**; 5(1), pp. 17-25.

25. Fogarty-Fairbanks, K.K.; Campbell, J.; Chase, C.L. Single Subcutaneous Dose of a Combination Vaccine Containing Bovine Herpes Virus-1 (BHV-1) Provides Protection Against an Intranasal BHV-1 Challenge 72 Hours Later. *Proceedings of the 2004 Annual Convention of the American Association of Bovine Practitioners*, **2004**; 37, pp. 289.
26. MacGregor, S.; Wray, M.I. The effect of bovine respiratory syncytial virus vaccination on health, feedlot performance and carcass characteristics of feeder cattle. *Bovine Practitioner*, **2004**; 38(2), pp. 162-170.
27. Plummer, P.J.; Rohrbach, B.W.; Daugherty, R.A.; Daugherty, R.A.; Thomas, K.V.; Wilkes, R.P.; Duggan, F.E.; Kennedy, M.A. Effect of intranasal vaccination against bovine enteric coronavirus on the occurrence of respiratory tract disease in a commercial backgrounding feedlot. *J Am Vet Med Assoc*, **2004**; 225(5), pp. 726-731.
28. Plummer, P. Effect of intranasal vaccination against bovine enteric coronavirus on the occurrence of respiratory disease in a commercial backgrounding feedlot. *Journal of Veterinary Internal Medicine*, **2004**; 18(3), pp. 397.
29. Ellis, J.A.; West, K.H.; Waldner, C.; Rhodes, C. Efficacy of a saponin-adjuvanted inactivated respiratory syncytial virus vaccine in calves. *CAN. VET. J.*, **2005**; 46(2), pp. 155-162.
30. Ellis, J.; Waldner, C.; Rhodes, C.; Van, R. Longevity of protective immunity to experimental bovine herpesvirus-1 infection following inoculation with a combination modified-live virus vaccine in beef calves. *Journal of the American Veterinary Medical Association*, **2005**; 227(1), pp. 123-128.
31. Vangeel, I.; Antonis, A.F.G.; Fluess, M.; Riegler, L.; Peters, A.R.; Harmeyer, S.S. Efficacy of a modified live intranasal bovine respiratory syncytial virus vaccine in 3-week-old calves experimentally challenged with BRSV. *Vet. J.*, **2007**; 174(3), pp. 627-635.
32. Bryant, T.C.; Nichols, J.R.; Adams, J.R.; Farmer, T.D.; Miies, D.G. Effect of tilmicosin alone or in combination with Mannheimia haemolytica toxoid administered at initial feedlot processing on morbidity and mortality of high-risk calves. *Bovine Practitioner*, **2008**; 42(1), pp. 50-54.
33. Bryant, T.C.; Rogers, K.C.; Stone, N.D.; Miles, D.G. Effect of viral respiratory vaccine treatment on performance, health and carcass traits of auction-origin feeder steers. *Bovine Practitioner*, **2008**; 42(1), pp. 98-103.
34. Cho, Y.S.; Lee, H.S.; Lim, S.K.; Joo, Y.S.; Kim, J.M.; Kim, J.H. Safety and efficacy testing of a novel multivalent bovine bacterial respiratory vaccine composed of five bacterins and two immunogens. *J. Vet. Med. Sci.*, **2008**; 70(9), pp. 959-964.
35. Step, D.L.; Krehbiel, C.R.; DePra, H.A.; Cranston, J.J.; Fulton, R.W.; Kirkpatrick, J.G.; Gill, D.R.; Payton, M.E.; Montelongo, M.A.; Confer, A.W. Effects of commingling beef calves from different sources and weaning protocols during a forty-two-day receiving period on performance and bovine respiratory disease. *J Anim Sci*, **2008**; 86(11), pp. 3146-3158.
36. Stilwell, G.; Matos, M.; Carolino, N.; Lima, M.S. Effect of a quadrivalent vaccine against respiratory virus on the incidence of respiratory disease in weaned beef calves. *Prev Vet Med*, **2008**; 85(3-4), pp. 151-157.
37. Wildman, B.K.; Perrett, T.; Abutarbush, S.M.; Guichon, P.T.; Pittman, T.J.; Booker, C.W.; Schunicht, O.C.; Fenton, R.K.; Jim, G.K. A comparison of 2 vaccination programs in feedlot calves at ultra-high risk of developing undifferentiated fever/bovine respiratory disease. *Can Vet J*, **2008**; 49(5), pp. 463-472.
38. Assié, S.; Seegers, H.; Makoschey, B.; Désirébousquié, L.; Bareille, N. Exposure to pathogens and incidence of respiratory disease in young bulls on their arrival at fattening operations in France. *Vet. Rec.*, **2009**; 165(7), pp. 195-199.

39. Ayalew, S.; Step, D.L.; Montelongo, M.; Confer, A.W. Intranasal vaccination of calves with Mannheimia haemolytica chimeric protein containing the major surface epitope of outer membrane lipoprotein PlpE, the neutralizing epitope of leukotoxin, and cholera toxin subunit B. *Vet. Immunol. Immunopathol.*, **2009**; 132(2-4), pp. 295-302.
40. Ellis, J.A.; Gow, S.E.; Goji, N.; Jones, C.; Workman, A.; Henderson, G.; Rhodes, C.; Alaniz, G.; Meinert, T.R.; Tucker, C.M. Efficacy of a combination viral vaccine for protection of cattle against experimental infection with field isolates of bovine herpesvirus-1. *Journal of the American Veterinary Medical Association*, **2009**; 235(5), pp. 563-572.
41. Razzaque, M.A.; Al-Mutawa, T.; Abbas, S.; Bedair, M. Performance of pre-weaned dairy calves under hot arid environment: effects of immunoglobulins and age on diseases and mortality. *American Journal of Applied Sciences*, **2009**; 6(11), pp. 1885-1891.
42. Rogers, K.C.; Portillo, T.A.; Smialek, D.E.; Miles, D.G.; Lehenbauer, T.W.; Smyth, R. A Comparison of Two Mannheimia haemolytica Vaccination Strategies in Freshly Weaned Southeastern Feedlot Heifers. *Bovine Practitioner*, **2009**; 43(1), pp. 27-31.
43. Step, D.L.; Krehbiel, C.R.; Burciaga-Robles, L.O.; Holland, B.R.; Fulton, R.W.; Confer, A.W.; Bechtol, D.T.; Brister, D.L.; Hutcheson, J.R.; Newcomb, H.L. Comparison of single vaccination versus revaccination with a modified-live virus vaccine containing bovine herpesvirus-1, bovine viral diarrhea virus (types la and 2a), parainfluenza type 3 virus, and bovine respiratory syncytial virus in the prevention of bovine respiratory disease in cattle. *Journal of the American Veterinary Medical Association*, **2009**; 235(5), pp. 580-587.
44. Vangeel, I.; Ioannou, F.; Riegler, L.; Salt, J.S.; Harmeyer, S.S. Efficacy of an intranasal modified live bovine respiratory syncytial virus and temperature-sensitive parainfluenza type 3 virus vaccine in 3-week-old calves experimentally challenged with PI3V. *Vet. J.*, **2009**; 179(1), pp. 101-108.
45. Wildman, B.K.; Jim, G.K.; Perrett, T.; Schunicht, O.C.; Hannon, S.J.; Fenton, R.K.; Abutarbush, S.M.; Booker, C.W. A Comparison of Two Multivalent Viral Vaccine Programs in Feedlot Calves at High Risk of Developing Undifferentiated Fever/Bovine Respiratory Disease. *Bovine Practitioner*, **2009**; 43(2), pp. 130-139.
46. Ellis, J.A.; Gow, S.P.; Goji, N. Response to experimentally induced infection with bovine respiratory syncytial virus following intranasal vaccination of seropositive and seronegative calves. *Journal of the American Veterinary Medical Association*, **2010**; 236(9), pp. 991-999.
47. Hanzlicek, G.A.; White, B.J.; Renter, D.G.; Blasi, D.A. A field study evaluating health, performance, and behavior differences in crossbred beef calves administered different vaccine-parasiticide product combinations. *VACCINE*, **2010**; 28(37), pp. 5998-6005.
48. Bryant, T.C.; Nichols, J.R.; Rogers, K.C.; Farmer, T.D.; Miles, D.G.; Campbell, J.; Richeson, J.T. Effect of Trivalent Modified-Live Virus Respiratory Vaccine on Performance, Health, and Carcass Traits of Lightweight Feeder Steers. *Bovine Practitioner*, **2011**; 45(2), pp. 124-130.
49. Windeyer, M.C.; Leslie, K.E.; Godden, S.M.; Hodgins, D.C.; Lissemore, K.D.; LeBlanc, S.J. The effects of viral vaccination of dairy heifer calves on the incidence of respiratory disease, mortality, and growth. *Journal of Dairy Science*, **2012**; 95(11), pp. 6731-6739.
50. Mulongo, M.; Prysliak, T.; Perez-Casal, J. Vaccination of feedlot cattle with extracts and membrane fractions from two Mycoplasma bovis isolates results in strong humoral immune responses but does not protect against an experimental challenge. *VACCINE*, **2013**; 31(10), pp. 1406-1412.
51. Poe, K.D.; Beck, P.A.; Richeson, J.T.; Gadberry, M.S.; Kegley, E.B.; Hess, T.W.; Hubbell, D.S. Effects of respiratory vaccination timing and growth-promoting implant on health, performance, and immunity of high-risk, newly received stocker cattle. *Professional Animal Scientist*, **2013**; 29(4), pp. 413-419.

52. Prysliak, T.; van der Merwe, J.; Perez-Casal, J. Vaccination with recombinant Mycoplasma bovis GAPDH results in a strong humoral immune response but does not protect feedlot cattle from an experimental challenge with M. bovis. *Microb Pathog*, **2013**; 55, pp. 1-8.
53. Zhang, R.; Han, X.; Chen, Y.; Mustafa, R.; Qi, J.; Chen, X.; Hu, C.; Chen, H.; Guo, A. Attenuated Mycoplasma bovis strains provide protection against virulent infection in calves. *VACCINE*, **2014**; 32(25), pp. 3107-3114.
54. Rogers, K.C.; Miles, D.G.; Hughes, H.D.; Renter, D.G.; Woodruff, J.; Zuidhof, S. Effect of initial respiratory viral-bacterial combination vaccine on performance, health, and carcass traits of auction-market derived feedlot heifers. *Bovine Practitioner*, **2015**; 49(1), pp. 43-47.
55. Dudek, K.; Bednarek, D.; Ayling, R.D.; Kycko, A.; Szacawa, E.; Karpińska, T.A. An experimental vaccine composed of two adjuvants gives protection against Mycoplasma bovis in calves. *VACCINE*, **2016**; 34(27), pp. 3051-3058.
56. Madampage, C.A.; Wilson, D.; Townsend, H.; Crockford, G.; Rawlyk, N.; Dent, D.; Evans, B.; Van Donkersgoed, J.; Dorin, C.; Potter, A. Cattle immunized with a recombinant subunit vaccine formulation exhibits a trend towards protection against Histophilus somni bacterial challenge. *PLoS ONE*, **2016**; 11(8).
57. Hause, B.M.; Huntimer, L.; Falkenberg, S.; Henningson, J.; Lechtenberg, K.; Halbur, T. An inactivated influenza D virus vaccine partially protects cattle from respiratory disease caused by homologous challenge. *VET. MICROBIOL.*, **2017**; 199, pp. 47-53.
58. White, B.J.; Theurer, M.E.; Goehl, D.R.; Thompson, P. Effect of modified-live bovine viral diarrhea virus type 2 vaccine on performance, health, temperature, and behavior response in high-risk beef heifer calves. *Bovine Practitioner*, **2017**; 51(1), pp. 38-47.
59. Griffin, C.M.; Scott, J.A.; Karisch, B.B.; Woolums, A.R.; Blanton, J.R.; Kaplan, R.M.; Epperson, W.B.; Smith, D.R. A randomized controlled trial to test the effect of on-arrival vaccination and deworming on stocker cattle health and growth performance. *Bov Pract (Stillwater)*, **2018**; 52(1), pp. 26-33.
60. Nagai, K.; Otomaru, K.; Ogawa, R.; Oishi, S.; Wataya, K.; Honkawa, Y.; Iwamoto, Y.; Ando, T.; Hyakutake, K.; Shirahama, H.; Habiby, G.; Kubota, C. Effect of combined vaccination for pasteurella multocida, mannheimia haemolytica, and Histophilus somni to prevent respiratory diseases in young Japanese black calves in the field. *J. Vet. Med. Sci.*, **2019**; 81(9), pp. 1355-1358.
61. Masset, N.; Meurens, F.; Marie, M.; Lesage, P.; Lehébel, A.; Brisseau, N.; Assié, S. Effectiveness of two intranasal vaccines for the control of bovine respiratory disease in newborn beef calves: A randomized non-inferiority multicentre field trial. *Vet. J.*, **2020**; 263.
62. Munoz, V.I.; Samuelson, K.L.; Tomczak, D.J.; Seiver, H.A.; Smock, T.M.; Richeson, J.T. Comparative efficacy of metaphylaxis with tulathromycin and pentavalent modified-live virus vaccination in high-risk, newly received feedlot cattle. *Applied Animal Science*, **2020**; 36(6), pp. 799-807.
63. Sandelin, A.; Härtel, H.; Seppä-Lassila, L.; Kaartinen, L.; Rautala, H.; Soveri, T.; Simojoki, H. Field trial to evaluate the effect of an intranasal respiratory vaccine protocol on bovine respiratory disease incidence and growth in a commercial calf rearing unit. *BMC Vet Res*, **2020**; 16(1), pp. 73.
64. Cusack, P.M.V.; Bergman, E.L.; Hay, K.E.; Morton, J.M. Health and production effects of killed vaccines against Mannheimia haemolytica, bovine viral diarrhoea virus and bovine herpesvirus 1, in locally backgrounded feedlot cattle. *Austr. Vet. J.*, **2021**; 99(1-2), pp. 24-31.

Nutritional supplementation:

1. Cole, N.A.; Purdy, C.W.; Hutcheson, D.P. Influence of yeast culture on feeder calves and lambs. *J Anim Sci*, **1992**; 70(6), pp. 1682-1690.
2. Moonsie-Shageer, S.; Mowat, D.N. Effect of level of supplemental chromium on performance, serum constituents, and immune status of stressed feeder calves. *J Anim Sci*, **1993**; 71(1), pp. 232-238.
3. Carter, J.N.; Meredith, G.L.; Montelongo, M.; Gill, D.R.; Krehbiel, C.R.; Payton, M.E.; Confer, A.W. Relationship of vitamin E supplementation and antimicrobial treatment with acute-phase protein responses in cattle affected by naturally acquired respiratory tract disease. *Am J Vet Res*, **2002**; 63(8), pp. 1111-1117.
4. Loneragan, G.H.; Morley, P.S.; Wagner, J.J.; Mason, G.L.; Yost, G.S.; Thoren, M.A.; Wittum, T.E.; Bray, T.M. Effects of feeding aspirin and supplemental vitamin E on plasma concentrations of 3-methylindole, 3-methyleneindolenine-adduct concentrations in blood and pulmonary tissues, lung lesions, and growth performance in feedlot cattle. *Am J Vet Res*, **2002**; 63(12), pp. 1641-1647.
5. Rivera, J.D.; Duff, G.C.; Galyean, M.L.; Walker, D.A.; Nunnery, G.A. Effects of supplemental vitamin E on performance, health, and humoral immune response of beef cattle. *Journal of Animal Science*, **2002**; 80(4), pp. 933-941.
6. Berry, B.A.; Krehbiel, C.R.; Confer, A.W.; Gill, D.R.; Smith, R.A.; Montelongo, M. Effects of dietary energy and starch concentrations for newly received feedlot calves: I. Growth performance and health. *J Anim Sci*, **2004**; 82(3), pp. 837-844.
7. Carter, J.N.; Gill, D.R.; Krehbiel, C.R.; Confer, A.W.; Smith, R.A.; Lalman, D.L.; Claypool, P.L.; McDowell, L.R. Vitamin E supplementation of newly arrived feedlot calves. *J Anim Sci*, **2005**; 83(8), pp. 1924-1932.
8. Clark, J.H.; Olson, K.C.; Schmidt, T.B.; Larson, R.L.; Ellersieck, M.R.; Alkire, D.O.; Meyer, D.L.; Rentfrow, G.K.; Carr, C.C. Effects of Respiratory Disease Risk and a Bolus Injection of Trace Minerals at Receiving on Growing and Finishing Performance by Beef Steers. *Professional Animal Scientist*, **2006**; 22(3), pp. 245-251.
9. Whitney, T.R.; Duff, G.C.; Collins, J.K.; Schafer, D.W.; Hallford, D.M. Effects of diet for early-weaned crossbred beef steers on metabolic profiles and febrile response to an infectious bovine herpesvirus-1 challenge. *Livestock Science*, **2006**; 101(1-3), pp. 1-9.
10. Corrigan, M.E.; Drouillard, J.S.; Spire, M.F.; Mosier, D.A.; Minton, J.E.; Higgins, J.J.; Loe, E.R.; Depenbusch, B.E.; Fox, J.T. Effects of melengestrol acetate on the inflammatory response in heifers challenged with Mannheimia haemolytica. *J Anim Sci*, **2007**; 85(7), pp. 1770-1779.
11. Keyser, S.A.; McMeniman, J.P.; Smith, D.R.; MacDonald, J.C.; Galyean, M.L. Effects of Saccharomyces cerevisiae subspecies boulardii CNCM I-1079 on feed intake by healthy beef cattle treated with florfenicol and on health and performance of newly received beef heifers. *J Anim Sci*, **2007**; 85(5), pp. 1264-1273.
12. Stanford, K.; McAllister, T.A.; Ayroud, M.; Bray, T.M.; Yost, G.S. Acute interstitial pneumonia in feedlot cattle: effects of feeding feather meal or vitamin E. *Can J Vet Res*, **2007**; 71(2), pp. 152-156.
13. Cusack, P.M. Effects of a dietary complex of humic and fulvic acids (FeedMAX 15) on the health and production of feedlot cattle destined for the Australian domestic market. *Aust Vet J*, **2008**; 86(1-2), pp. 46-49.
14. Farran, T.B.; Reinhardt, C.D.; Blasi, D.A.; Minton, J.E.; Elsasser, T.H.; Higgins, J.J.; Drouillard, J.S. Source of dietary lipid may modify the immune response in stressed feeder cattle. *J Anim Sci*, **2008**; 86(6), pp. 1382-1394.

15. Perrett, T.; Wildman, B.K.; Abutarbush, S.M.; Pittman, T.J.; Jones, C.; Pollock, C.M.; Schunicht, O.C.; Guichon, P.T.; Jim, G.K.; Booker, C.W. A comparison of two Mannheimia haemolytica immunization programs in feedlot calves at high risk of developing undifferentiated fever/bovine respiratory disease. *Bovine Practitioner*, **2008**; 42(1), pp. 64-75.
16. Perrett, T.; Wildman, B.K.; Jim, G.K.; Vogstad, A.R.; Fenton, R.K.; Hannon, S.J.; Schunicht, O.C.; Abutarbush, S.M.; Booker, C.W. Evaluation of the efficacy and cost-effectiveness of melengestrol acetate in feedlot heifer calves in western Canada. *Vet Ther*, **2008**; 9(3), pp. 223-240.
17. Quinn, M.J.; Moore, E.S.; Thomson, D.U.; Depenbusch, B.E.; May, M.L.; Higgins, J.J.; Carter, J.F.; Drouillard, J.S. The effects of feeding flaxseed during the receiving period on morbidity, mortality, performance, and carcass characteristics of heifers. *Journal of Animal Science*, **2008**; 86(11), pp. 3054-3061.
18. Matsumoto, D.; Takagi, M.; Hasunuma, H.; Fushimi, Y.; Ohtani, M.; Sato, T.; Okamoto, K.; Shahada, F.; Tanaka, T.; Deguchi, E. Effects of Oral Administration of Difructose Anhydride III on Selected Health and Blood Parameters of Group-housed Japanese Black Calves during the Preweaning Period. *Asian-Australasian Journal of Animal Sciences*, **2009**; 22(12), pp. 1640-1647.
19. Covey, T.L.; Elam, N.E.; Carroll, J.A.; Wester, D.B.; Galyean, M.L. Supplemental selenium source in Holstein steers challenged with intranasal bovine infectious rhinotracheitis virus and in newly received beef heifers: performance, morbidity, antibody titers, and blood cell counts. *Professional Animal Scientist*, **2010**; 26(1), pp. 82-92.
20. Richeson, J.T.; Kegley, E.B. Effect of supplemental trace minerals from injection on health and performance of highly stressed, newly received beef heifers. *Professional Animal Scientist*, **2011**; 27(5), pp. 461-466.
21. Ponce, C.H.; Smith, D.R.; Schutz, J.S.; Galyean, M.L. Effects of receiving diets based on wet corn gluten feed on performance and morbidity of newly received beef heifers and in vitro fermentation. *Professional Animal Scientist*, **2012**; 28(2), pp. 213-220.
22. Ponce, C.H.; Schutz, J.S.; Elrod, C.C.; Anele, U.Y.; Galyean, M.L. Effects of dietary supplementation of a yeast product on performance and morbidity of newly received beef heifers. *Professional Animal Scientist*, **2012**; 28(6), pp. 618-622.
23. Hales, K.E.; Kraich, K.J.; Bondurant, R.G.; Meyer, B.E.; Luebbe, M.K.; Brown, M.S.; Cole, N.A.; MacDonald, J.C. Effects of glycerin on receiving performance and health status of beef steers and nutrient digestibility and rumen fermentation characteristics of growing steers. *Journal of Animal Science*, **2013**; 91(9), pp. 4277-4289.
24. Amat, S.; McKinnon, J.J.; Simko, E.; Hendrick, S. Evaluation of feeding corn or wheat dried distillers' grains with solubles on animal health of finishing feedlot steers. *Canadian Journal of Animal Science*, **2014**; 94(3), pp. 525-531.
25. Croft, I.; Clayton, E.; Cusack, P. Health and production of feedlot cattle following supplementation with urea-molasses in starter pens. *Aust Vet J*, **2014**; 92(5), pp. 166-170.
26. Finck, D.N.; Ribeiro, F.R.B.; Burdick, N.C.; Parr, S.L.; Carroll, J.A.; Young, T.R.; Bernhard, B.C.; Corley, J.R.; Estefan, A.G.; Rathmann, R.J.; Johnson, B.J. Yeast supplementation alters the performance and health status of receiving cattle. *Professional Animal Scientist*, **2014**; 30(3), pp. 333-341.
27. Ryan, A.W.; Kegley, E.B.; Hawley, J.; Powell, J.G.; Hornsby, J.A.; Reynolds, J.L.; Laudert, S.B. Supplemental trace minerals (zinc, copper, and manganese) as sulfates, organic amino acid complexes, or hydroxy trace-mineral sources for shipping-stressed calves. *Prof Anim Sci*, **2015**; 31(4), pp. 333-341.

28. Roberts, S.L.; May, N.D.; Brauer, C.L.; Gentry, W.W.; Weiss, C.P.; Jennings, J.S.; Richeson, J.T. Effect of injectable trace mineral administration on health, performance, and vaccine response of newly received feedlot cattle. *Professional Animal Scientist*, **2016**; 32(6), pp. 842-848.
29. Wilson, B.K.; Vazquez-Anon, M.; Step, D.L.; Moyer, K.D.; Haviland, C.L.; Maxwell, C.L.; Neill, C.F.; Gifford, C.A.; Krehbiel, C.R.; Richards, C.J. Effect of copper, manganese, and zinc supplementation on the performance, clinical signs, and mineral status of calves following exposure to bovine viral diarrhea virus type 1b and subsequent Mannheimia haemolytica infection. *Journal of Animal Science*, **2016**; 94(3), pp. 1123-1140.
30. Lippolis, K.D.; Cooke, R.F.; Schumaher, T.; Brandão, A.P.; Silva, L.G.T.; Schubach, K.M.; Marques, R.S.; Bohnert, D.W. Physiologic, health, and performance responses of beef steers supplemented with an immunomodulatory feed ingredient during feedlot receiving. *J Anim Sci*, **2017**; 95(11), pp. 4945-4957.
31. Schubach, K.M.; Cooke, R.F.; Silva, L.G.T.; Brandão, A.P.; Lippolis, K.D.; Marques, R.S.; Bohnert, D.W. Supplementing a yeast-derived product to enhance productive and health responses of feeder steers. *Translational Animal Science*, **2017**; 1(Suppl. 1), pp. 132-137.
32. De Souza, K.; Cooke, R.; Schubach, K.; Brandão, A.; Schumaher, T.; Prado, I.; Marques, R.; Bohnert, D. Performance, health and physiological responses of newly weaned feedlot cattle supplemented with feed-grade antibiotics or alternative feed ingredients. *animal*, **2018**; 12(12), pp. 2521-2528.
33. Deters, E.L.; Stokes, R.S.; Genther-Schroeder, O.N.; Hansen, S.L. Effects of a Saccharomyces cerevisiae fermentation product in receiving diets of newly weaned beef steers. II. Digestibility and response to a vaccination challenge. *Journal of Animal Science*, **2018**; 96(9), pp. 3906-3915.
34. Pukrop, J.R.; Brennan, K.M.; Funnell, B.J.; Schoonmaker, J.P. Effect of a hydrolyzed mannan- and glucan-rich yeast fraction on performance and health status of newly received feedlot cattle. *Journal of Animal Science*, **2018**; 96(9), pp. 3955-3966.
35. Schubach, K.M.; Cooke, R.; Souza, K.A.; Schumaher, T.F.; Silva, N.S.; Bohnert, D.W.; Marques, R. Performance, Health, and Physiological Responses of Newly-Weaned Cattle Supplemented with Feed Grade Antibiotics or Alternative Feed Additives during Feedlot Receiving. *Journal of Animal Science*, **2018**; 96(Suppl. 1), pp. 22.
36. Silva, L.G.T.; Cooke, R.F.; Schubach, K.M.; Brandão, A.P.; Marques, R.S.; Schumaher, T.F.; Moriel, P.; Bohnert, D.W. Supplementing a yeast-derived product to enhance productive and health responses of beef steers. *Animal*, **2018**; 12(8), pp. 1576-1583.
37. Spore, T.J.; Montgomery, S.P.; Titgemeyer, E.C.; Hanzlicek, G.A.; Vahl, C.I.; Nagaraja, T.G.; Cavalli, K.T.; Hollenbeck, W.R.; Wahl, R.A.; Blasi, D.A. Effects of dietary energy level and intake of corn by-product-based diets on newly received growing cattle: Antibody production, acute phase protein response, stress, and immunocompetency of healthy and morbid animals. *Journal of Animal Science*, **2018**; 96(4), pp. 1474-1483.
38. Woolsoncroft, M.A.; Youngers, M.E.; McPhillips, L.J.; Lockard, C.G.; Haviland, C.L.; DeSocio, E.S.; Ryan, W.R.; Richards, C.J.; Wilson, B.K. Effects of exercise and roughage source on the health and performance of receiving beef calves. *Professional Animal Scientist*, **2018**; 34(2), pp. 183-191.
39. De Sousa, O.A.; Cooke, R.F.; Brandão, A.P.; Schubach, K.M.; Schumaher, T.F.; Bohnert, D.W.; Marques, R.S. Productive and physiological responses of feeder cattle supplemented with Yucca schidigera extract during feedlot receiving. *J Anim Sci*, **2019**; 97(1), pp. 208-219.

40. Deters, E.L.; Hansen, S.L. Vitamin E supplementation strategies during feedlot receiving: effects on beef steer performance, antibody response to vaccination, and antioxidant defense1. *J Anim Sci*, **2019**; 97(10), pp. 4362-4369.
41. Smock, T.M.; Samuelson, K.; Seiver, H.A.; Hergenreder, J.; Rounds, W. Effects of Bacillus subtilis PB6 and/or chromium propionate supplementation on health, performance, and blood parameters of high-risk cattle during the feedlot receiving period. *Journal of Animal Science*, **2019**; 97(Suppl. 3), pp. 231.
42. Spore, T.J.; Montgomery, S.P.; Titgemeyer, E.C.; Hanzlicek, G.A.; Vahl, C.I.; Nagaraja, T.G.; Cavalli, K.T.; Hollenbeck, W.R.; Wahl, R.A.; Blasi, D.A. Effects of a high-energy programmed feeding protocol on nutrient digestibility, health, and performance of newly received growing beef cattle. *Applied Animal Science*, **2019**; 35(4), pp. 397-407.
43. Theurer, M.E.; Fox, J.T.; Aguilar, A.; Nielsen, H.; Simpson, J.; Lawrence, T.E. Effect of live yeast (Saccharomyces cerevisiae boulardii CNCM I-1079) feed additive on health and growth parameters of high-risk heifers in a commercial feedlot. *Bovine Practitioner*, **2019**; 53(2), pp. 117-127.
44. Deikun, L.L.; Habing, G.G.; Quigley, J.D.; Proudfoot, K.L. Health and growth of veal calves provided a fatty acid supplement and a dry teat. *Journal of Dairy Science*, **2020**; 103(5), pp. 4633-4642.
45. Galyen, W.L.; Beck, P.A.; Hess, T.W.; Hubbell, D.S.; Gadberry, M.S.; Kegley, E.B.; Cravey, M.; Powell, J.G. Effects of bambermycin with amprolium or monensin on beef cattle growth performance, coccidia infection, and bovine respiratory disease morbidity during receiving. *Applied Animal Science*, **2020**; 36(3), pp. 423-429.
46. Smock, T.M.; Samuelson, K.L.; Hergenreder, J.E.; Whitney Rounds, P.; Richeson, J.T. Effects of Bacillus subtilis PB6 and/or chromium propionate supplementation on clinical health, growth performance, and carcass traits of high-risk cattle during the feedlot receiving and finishing periods. *Translational Animal Science*, **2020**; 4(3), pp. 1-12.
47. Virmond, M.P.; Rossi, P.S.; Antunes, A.V.; Mattei, R.I.; Schllemer, N.R.; Thomaz, G.R.; Garbossa, G.; Neumann, M.; Bertagnon, H.G. Fresh yeast additives improve immune parameters and reduce respiratory disease in heifers finished in feedlots. *Semin. Cienc. Agrar.*, **2020**; 41(6), pp. 3177-3188.

Animal characteristics and genetic traits:

1. Muggli-Cockett, N.E.; Cundiff, L.V.; Gregory, K.E. Genetic analysis of bovine respiratory disease in beef calves during the first year of life. *J Anim Sci*, **1992**; 70(7), pp. 2013-2019.
2. Ryan, A.M.; Hutcheson, D.P.; Womack, J.E. Type-I interferon genotypes and severity of clinical disease in cattle inoculated with bovine herpesvirus 1. *American Journal of Veterinary Research*, **1993**; 54(1), pp. 73-79.
3. Norström, M.; Skjerve, E.; Jarp, J. Risk factors for epidemic respiratory disease in Norwegian cattle herds. *Prev. Vet. Med.*, **2000**; 44(1-2), pp. 87-96.
4. Assié, S.; Seegers, H.; Beaudeau, F. Incidence of respiratory disorders during housing in non-weaned Charolais calves in cow-calf farms of Pays de la Loire (western France). *Prev. Vet. Med.*, **2004**; 63(3-4), pp. 271-282.
5. Lago, A.; McGuirk, S.M.; Bennett, T.B.; Cook, N.B.; Nordlund, K.V. Calf respiratory disease and pen microenvironments in naturally ventilated calf barns in winter. *J Dairy Sci*, **2006**; 89(10), pp. 4014-4025.
6. Snowder, G.D.; Vleck, L.D.v.; Cundiff, L.V.; Bennett, G.L. *Genetic parameters for respiratory disease in feedlot beef cattle*, **2006**; Instituto Prociência: Minas Gerais. p. 15-14.

7. Snowder, G.D.; Van Vleck, L.D.; Cundiff, L.V.; Bennett, G.L. Bovine respiratory disease in feedlot cattle: environmental, genetic, and economic factors. *J Anim Sci*, **2006**; 84(8), pp. 1999-2008.
8. Cusack, P.M.; McMeniman, N.P.; Lean, I.J. Feedlot entry characteristics and climate: their relationship with cattle growth rate, bovine respiratory disease and mortality. *Aust Vet J*, **2007**; 85(8), pp. 311-316.
9. Heringstad, B.; Change, Y.M.; Gianola, D.; Østerås, O. Short communication: Genetic analysis of respiratory disease in Norwegian Red calves. *Journal of Dairy Science*, **2008**; 91(1), pp. 367-370.
10. Schneider, M.J.; Tait, R.G.; Jr, R.; M. V, B.; W. D, R. Evaluation of fixed sources of variation and estimation of genetic parameters for incidence of bovine respiratory disease in preweaned calves and feedlot cattle. *J Anim Sci*, **2010**; 88(4), pp. 1220-1228.
11. Fulton, R.W.; Cook, B.J.; Blood, K.S.; Confer, A.W.; Payton, M.E.; Step, D.L.; Saliki, J.T.; Burge, L.J.; Welsh, R.D. Immune Response to Bovine Respiratory Disease Vaccine Immunogens in Calves at Entry to Feedlot and Impact on Feedlot Performance. *Bovine Practitioner*, **2011**; 45(1), pp. 1-12.
12. Brscic, M.; Leruste, H.; Heutinck, L.F.; Bokkers, E.A.; Wolthuis-Fillerup, M.; Stockhofe, N.; Gottardo, F.; Lensink, B.J.; Cozzi, G.; Van Reenen, C.G. Prevalence of respiratory disorders in veal calves and potential risk factors. *J Dairy Sci*, **2012**; 95(5), pp. 2753-2764.
13. Cernicchiaro, N.; White, B.J.; Renter, D.G.; Babcock, A.H.; Kelly, L.; Slattery, R. Associations between the distance traveled from sale barns to commercial feedlots in the United States and overall performance, risk of respiratory disease, and cumulative mortality in feeder cattle during 1997 to 2009. *J Anim Sci*, **2012**; 90(6), pp. 1929-1939.
14. Van Eenennaam, A.; Neibergs, H.; Seabury, C.; Taylor, J.; Wang, Z.; Scraggs, E.; Schnabel, R.D.; Decker, J.; Wojtowicz, A.; Aly, S.; Davis, J.; Blanchard, P.; Crossley, B.; Rossitto, P.; Lehenbauer, T.; Hagevoort, R.; Chavez, E.; Neibergs, J.S.; Womack, J.E. Results of the BRD CAP project: progress toward identifying genetic markers associated with BRD susceptibility. *Anim Health Res Rev*, **2014**; 15(2), pp. 157-160.
15. Neupane, M.; Kiser, J.N.; Seabury, C.M.; Taylor, J.F.; Womack, J.E.; The Bovine Respiratory Disease Complex Coordinated Agricultural Project Research Team, N. Genetic approaches to identify genomic regions associated with decreased susceptibility to bovine respiratory disease complex. *Annual Convention of the American Association of Bovine Practitioners*, **2015**; 48, pp. 148-153.
16. Cockrum, R.R.; Speidel, S.E.; Salak-Johnson, J.L.; Chase, C.C.; Peel, R.K.; Weaber, R.L.; Loneagan, G.H.; Wagner, J.J.; Boddhireddy, P.; Thomas, M.G.; Prayaga, K.; DeNise, S.; Enns, R.M. Genetic parameters estimated at receiving for circulating cortisol, immunoglobulin G, interleukin 8, and incidence of bovine respiratory disease in feedlot beef steers. *J Anim Sci*, **2016**; 94(7), pp. 2770-2778.
17. Hay, K.E.; Morton, J.M.; Mahony, T.J.; Clements, A.C.; Barnes, T.S. Associations between animal characteristic and environmental risk factors and bovine respiratory disease in Australian feedlot cattle. *Prev Vet Med*, **2016**; 125, pp. 66-74.
18. Hay, K.E.; Morton, J.M.; Schibrowski, M.L.; Clements, A.C.; Mahony, T.J.; Barnes, T.S. Associations between prior management of cattle and risk of bovine respiratory disease in feedlot cattle. *Prev Vet Med*, **2016**; 127, pp. 37-43.
19. Lipkin, E.; Strillacci, M.G.; Eitam, H.; Yishay, M.; Schiavini, F.; Soller, M.; Bagnato, A.; Shabtay, A. The Use of Kosher Phenotyping for Mapping QTL Affecting Susceptibility to Bovine Respiratory Disease. *PLoS ONE*, **2016**; 11(4), pp. e0153423.

20. Murray, G.M.; Cassidy, J.P.; Clegg, T.A.; Tratalos, J.A.; McClure, J.; O'Neill, R.G.; Sammin, D.J.; Casey, M.J.; McElroy, M.; Earley, B.; Bourke, N.; More, S.J. A retrospective epidemiological analysis of risk factors for a primary necropsy diagnosis of bovine respiratory disease. *Prev Vet Med*, **2016**; 132, pp. 49-56.
21. Hay, K.E.; Morton, J.M.; Clements, A.C.A.; Mahony, T.J.; Barnes, T.S. Population-level effects of risk factors for bovine respiratory disease in Australian feedlot cattle. *Prev Vet Med*, **2017**; 140, pp. 78-86.
22. Pempek, J.; Trearchis, D.; Masterson, M.; Habing, G.; Proudfoot, K. Veal calf health on the day of arrival at growers in Ohio. *J Anim Sci*, **2017**; 95(9), pp. 3863-3872.
23. Neupane, M.; Kiser, J.N.; Neibergs, H.L. Gene set enrichment analysis of SNP data in dairy and beef cattle with bovine respiratory disease. *Anim Genet*, **2018**; 49(6), pp. 527-538.
24. Schibrowski, M.L.; Gibson, J.S.; Hay, K.E.; Mahony, T.J.; Barnes, T.S. Mycoplasma bovis and bovine respiratory disease: A risk factor study in Australian feeder cattle. *Prev Vet Med*, **2018**; 157, pp. 152-161.
25. Hoff, J.L.; Decker, J.E.; Schnabel, R.D.; Seabury, C.M.; Neibergs, H.L.; Taylor, J.F. QTL-mapping and genomic prediction for bovine respiratory disease in U.S. Holsteins using sequence imputation and feature selection. *BMC Genomics*, **2019**; 20(1), pp. 555.
26. Sasaki, Y.; Hashimoto, K.; Iki, Y.; Anan, T.; Hayashi, J.; Uematsu, M. Associations between calf factors of Japanese Black calves arriving at a backgrounding operation and bovine respiratory disease. *Prev. Vet. Med.*, **2020**; 182.
27. Scott, M.A.; Woolums, A.R.; Swiderski, C.E.; Perkins, A.D.; Nanduri, B.; Smith, D.R.; Karisch, B.B.; Epperson, W.B.; Blanton, J.R. Whole blood transcriptomic analysis of beef cattle at arrival identifies potential predictive molecules and mechanisms that indicate animals that naturally resist bovine respiratory disease. *PLoS ONE*, **2020**; 15(1), pp. e0227507.

Miscellaneous other interventions and risk factors:

1. Dubeski, P.L.; D'Offay, J.M.; Owens, F.N. Effect of B-vitamin injections on virus infection and immunity in feed-restricted weaned beef calves. *Animal Science Research Report, Agricultural Experiment Station, Oklahoma State University*, **1992**(MP-136), pp. 251-257.
2. Jim, G.K.; Booker, C.W.; Guichon, P.T. Comparison of a combination of oxfendazole and fenthion versus ivermectin in feedlot calves. *Can Vet J*, **1992**; 33(9), pp. 599-604.
3. Mosier, D.A.; Simons, K.R.; Vestweber, J.G. Passive protection of calves with Pasteurella haemolytica antiserum. *American Journal of Veterinary Research*, **1995**; 56(10), pp. 1317-1321.
4. Bingham, H.R.; Morley, P.S.; Wittum, T.E.; Bray, T.M.; West, K.H.; Slemons, R.D.; Ellis, J.A.; Haines, D.M.; Levy, M.A.; Sarver, C.F.; Saville, W.J.; Cortese, V.S. Synergistic effects of concurrent challenge with bovine respiratory syncytial virus and 3-methylindole in calves. *Am J Vet Res*, **1999**; 60(5), pp. 563-570.
5. Cummins, J.M.; Guthrie, D.; Hutcheson, D.P.; Krakowka, S.; Rosenquist, B.D. Natural human interferon-alpha administered orally as a treatment of bovine respiratory disease complex. *J Interferon Cytokine Res*, **1999**; 19(8), pp. 907-910.
6. Bingham, H.R.; Wittum, T.E.; Morley, P.S.; Bray, T.M.; Sarver, C.F.; Queen, W.G.; Shulaw, W.P. Evaluation of the ability of orally administered aspirin to mitigate effects of 3-methylindole in feedlot cattle. *Am J Vet Res*, **2000**; 61(10), pp. 1209-1213.
7. Nicholas, R.A.J.; Ayling, R.D.; Stipkovits, L.P. An experimental vaccine for calf pneumonia caused by Mycoplasma bovis: Clinical, cultural, serological and pathological findings. *VACCINE*, **2002**; 20(29-30), pp. 3569-3575.

8. Montgomery, S.P.; Sindt, J.J.; Greenquist, M.A.; Loe, E.R.; Drouillard, J.S. Comparison of bovine transfer factor and tilmicosin phosphate: effects on health and growth performance of newly arrived feedlot heifers. *International Journal of Applied Research in Veterinary Medicine*, **2008**; 6(3), pp. 175-180.
9. Regev-Shoshani, G.; Church, J.S.; Cook, N.J.; Schaefer, A.L.; Miller, C. Prophylactic nitric oxide treatment reduces incidence of bovine respiratory disease complex in beef cattle arriving at a feedlot. *Res Vet Sci*, **2013**; 95(2), pp. 606-611.
10. Teixeira, A.G.V.; Lima, F.S.; Bicalho, M.L.S.; Kussler, A.; Lima, S.F.; Felippe, M.J.; Bicalho, R.C. Effect of an injectable trace mineral supplement containing selenium, copper, zinc, and manganese on immunity, health, and growth of dairy calves. *Journal of Dairy Science*, **2014**; 97(7), pp. 4216-4226.
11. Richeson, J.T.; Beck, P.A.; Hughes, H.D.; Hubbell, D.S.; Gadberry, M.S.; Kegley, E.B.; Powell, J.G.; Prouty, F.L. Effect of growth implant regimen on health, performance, and immunity of high-risk, newly received stocker cattle. *Journal of Animal Science*, **2015**; 93(8), pp. 4089-4097.
12. Bailey, E.A.; Jaeger, J.R.; Schmidt, T.B.; Waggoner, J.W.; Pacheco, L.A.; Thomson, D.U.; Olson, K.C. Effects of number of viral respiratory disease vaccinations during preconditioning on health, performance, and carcass merit of ranch-direct beef calves during receiving and finishing. *Professional Animal Scientist*, **2016**; 32(3), pp. 271-278.
13. Crepieux, T.; Miller, C.; Regev-Shoshani, G.; Schaefer, A.; Dorin, C.; Alexander, T.; Timsit, E. Randomized, non-inferiority trial comparing a nitric oxide releasing solution with a macrolide antibiotic for control of bovine respiratory disease in beef feedlot calves at high-risk of developing respiratory tract disease. *Res Vet Sci*, **2016**; 105, pp. 216-221.
14. Regev-Shoshani, G.; McMullin, B.; Nation, N.; Church, J.S.; Dorin, C.; Miller, C. Non-inferiority of nitric oxide releasing intranasal spray compared to sub-therapeutic antibiotics to reduce incidence of undifferentiated fever and bovine respiratory disease complex in low to moderate risk beef cattle arriving at a commercial feedlot. *Prev Vet Med*, **2017**; 138, pp. 162-169.
15. Timsit, E.; Workentine, M.; Crepieux, T.; Miller, C.; Regev-Shoshani, G.; Schaefer, A.; Alexander, T. Effects of nasal instillation of a nitric oxide-releasing solution or parenteral administration of tilmicosin on the nasopharyngeal microbiota of beef feedlot cattle at high-risk of developing respiratory tract disease. *Res Vet Sci*, **2017**; 115, pp. 117-124.
16. Engen, N.K.v.; Engelken, T.J.; Lockard, C.G.; Lakritz, J.; Cernicchiaro, N.; Wilson, B.K.; Krehbiel, C.R.; Coetzee, J.F. The effects of pretransportation or arrival meloxicam administration to calves entering the feedlot on morbidity, biomarkers, performance, and carcass characteristics. *Translational Animal Science*, **2019**; 3(2), pp. 620-632.
17. Wheat, W.; Chow, L.; Rozo, V.; Herman, J.; Brooks, K.S.; Colbath, A.; Hunter, R.; Dow, S. Non-specific protection from respiratory tract infections in cattle generated by intranasal administration of an innate immune stimulant. *PLoS ONE*, **2020**; 15(6).
